# Diet-induced obesity affects influenza disease severity and transmission dynamics in ferrets

**DOI:** 10.1126/sciadv.adk9137

**Published:** 2024-05-10

**Authors:** Victoria Meliopoulos, Rebekah Honce, Brandi Livingston, Virginia Hargest, Pamela Freiden, Lauren Lazure, Pamela H. Brigleb, Erik Karlsson, Heather Sheppard, E. Kaity Allen, David Boyd, Paul G. Thomas, Stacey Schultz-Cherry

**Affiliations:** ^1^ Department of Host-Microbe Interactions, St. Jude Children’s Research Hospital, Memphis, TN, USA.; ^2^ Veterinary Pathology Core, St. Jude Children’s Research Hospital, Memphis, TN, USA.; ^3^ Department of Immunology, St. Jude Children’s Research Hospital, Memphis, TN, USA.

## Abstract

Obesity, and the associated metabolic syndrome, is a risk factor for increased disease severity with a variety of infectious agents, including influenza virus. Yet, the mechanisms are only partially understood. As the number of people, particularly children, living with obesity continues to rise, it is critical to understand the role of host status on disease pathogenesis. In these studies, we use a diet-induced obese ferret model and tools to demonstrate that, like humans, obesity resulted in notable changes to the lung microenvironment, leading to increased clinical disease and viral spread to the lower respiratory tract. The decreased antiviral responses also resulted in obese animals shedding higher infectious virus for a longer period, making them more likely to transmit to contacts. These data suggest that the obese ferret model may be crucial to understanding obesity’s impact on influenza disease severity and community transmission and a key tool for therapeutic and intervention development for this high-risk population.

## Teaser

A diet-induced obese ferret model can elucidate mechanisms of influenza disease severity and transmission dynamics.

## INTRODUCTION

Obesity, and the associated metabolic syndrome (MetS), is a risk factor for increased disease severity with a variety of infectious agents, including influenza virus, severe acute respiratory syndrome coronavirus 2 (SARS-CoV-2), respiratory syncytial virus, and bacterial infection (*1*–*3*). Human cohort studies have reported that participants with a higher body mass index (BMI) are more likely to report symptoms and may have higher viral titers in exhaled breath (*4*, *5*), prolonged shed time resulting in a longer duration of illness, and increased susceptibility to certain strains of influenza virus (*6*, *7*). Yet, the mechanisms behind increased disease severity in obese individuals are only partially understood. As the number of people, particularly children, living with obesity continues to rise (*8*–*11*), it is critical to understand the role of host status on disease pathogenesis.

Genetic and diet-induced obese mouse models (*12*–*14*) have provided valuable insights into the impact of obesity on influenza disease, viral evolution, and therapeutic strategies (*15*–*18*). Yet, it is difficult to study many key aspects of disease, including viral transmission, in mice. Ferrets, however, can be infected with circulating human viruses without the need for adaptation, display similar clinical signs as humans, and can transmit virus through direct contact and via aerosols (*19*, *20*). We hypothesized that these animals could provide better clarity as to how obesity could affect influenza dynamics in human populations.

While it is possible to make transgenic ferrets (*21*, *22*), diet-induced obesity is a more accurate representation of human obesity. In these studies, we describe the development of the first diet-induced obese ferret model and the impact on influenza disease severity and transmission. Obese ferrets have increased waist circumference and additional visceral fat compared to control ferrets fed with a standard chow. They also had clinical parameters associated with development of MetS. When infected with influenza virus, obese ferrets experienced more severe clinical symptoms than control ferrets of healthy weight independent of viral strain. Lung injury and viral spread to the lower respiratory tract was enhanced. Further, we designed a panel of validated primers and probes targeting immune and lung function genes and found that obese ferrets had a dysregulated antiviral response in the lungs at baseline and after infection. Diet also influenced viral transmission. Obese ferrets were more likely to transmit an avian-like H9N2 influenza virus to other obese ferrets, while likelihood of transmission was equivalent when the transmitting ferret was on our control diet. Overall, our studies show that obesity in ferrets affects disease pathogenesis and transmission. This model and the ferret tools developed will be invaluable for better understanding the impact of obesity on disease pathogenesis, viral transmission, and evolution and will be crucial for assessing the impact of vaccines and antivirals on disease severity within this increasing population.

## RESULTS

### Obesity and associated MetS can be induced in ferrets

Male ferrets were maintained on a high calorie diet (obese) or a nutritionally balanced calorie restricted diet (control) for a period of 12 weeks as described in table S1. Ferrets in the obese group gained significantly more weight (fig. S1A). Waist circumference, which is strongly associated with increased risk of mortality in humans (*23*, *24*), abdominal adipose tissue, plasma leptin levels, skinfold fat, and overall BMI were elevated in the obese group (fig. S1, B to G). At the conclusion of the diet period, obese ferrets had developed biochemical hallmarks of MetS such as increased fasting glucose and total cholesterol (fig. S1, H and I). High-density lipoprotein (HDL) was reduced in obese ferrets, while low-density lipoprotein (LDL) was increased, indicating an unhealthy cholesterol balance (fig. S1, J to L) and that these are clinically associated with human obesity (*25*). Individual ferret measurements throughout the diet period are shown in fig. S2.

**Fig. 1.**
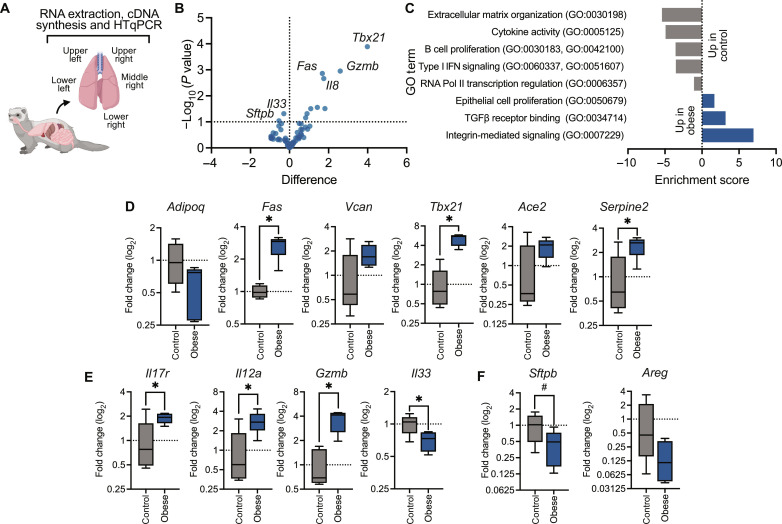
Obesogenic diet alters the pulmonary microenvironment at baseline. (**A**) After 12 weeks on diet, male obese and control ferrets were euthanized, and individual lobes of the lungs were resected. Tissues were homogenized, and RNA was extracted for high-throughput PCR. Each lobe was considered a separate sample. Illustration created with Biorender.com. (**B**) Volcano plot of differences in gene expression between control and obese ferrets, represented by −log_10_ of *p*-value against log_2_ fold change. (**C**) Database for Annotation, Visualization and Integrated Discovery enrichment analysis of up- and down-regulated genes in uninfected obese ferret lungs. Absolute value indicates enrichment score, with negative scores indicating relative down-regulation from uninfected control lungs. (**D** to **F**) Differences in pulmonary gene expression between control and obese ferrets at homeostasis. Fold expression was calculated via the 2^−ΔΔCt^ method. (D) Obesity-related markers are increased in obese ferrets. Expression of Fas cell surface death receptor (*Fas*, *P* = 0.0014), T-box transcription factor 21 (*Tbx21*, *P* = 0.0001), and serpin family E member 2 (*Serpine2*, *P* = 0.0277) was significantly increased. (E) Obese ferrets have significantly increased levels of the immune and antiviral defense genes interleukin-7 receptor (*Il7r*, *P* = 0.0484), interleukin-12A (*Il12a*, *P* = 0.0310), granzyme B (*Gzmb*, *P* = 0.0011), and interleukin-33 (*Il33*, *P* = 0.0484). (F) Decreased pulmonary surfactant (*Sftpb*, *P* = 0.0911) and amphiregulin (*Areg*) in obese ferrets. Significance was determined by unpaired *t* test. Data represent the average of five lung lobes per *n* = 1 ferret per group and one independent experiment of *n* = 1 per group. Samples were run in duplicate. Error bars represent minimum value to maximum value. *P < 0.05 and #*P* < 0.10. HTqPCR, high-throughput quantitative polymerase chain reaction; GO, Gene Ontology; TGFβ, transforming growth factor–β.

Obesity has been shown to affect the lung microenvironment and is associated with increased expression of pro-inflammatory cytokines and chemokines (*26*, *27*). Because the availability of ferret reagents is limited, we designed a panel of primers targeting lung function and immune response genes (table S2) and validated as described in Materials and Methods. We normalized gene expression in the uninfected obese ferrets to the corresponding gene expression in the uninfected control to compare status of the lung microenvironment at baseline (Fig. 1A). Even at baseline, gene expression differed in obese compared to control ferrets; globally, obese ferrets show decreased ability to induce antiviral responses and increased inflammatory signaling as assessed by gene ontology (Fig. 1, B and C). Obese ferrets had decreased adiponectin (*Adipoq*) levels, similar to obese humans (*28*, *29*). Fas cell surface death receptor (*Fas*), versican (*Vcan*), T-box transcription factor 21 (*Tbx21*), angiotensin I converting enzyme (*Ace*), and serpin family E member 2 (*Serpine2*), metabolic markers associated with obesity (*30*–*35*), were increased in the obese group (Fig. 1D). In addition, the baseline pulmonary environment was pro-inflammatory. Elevated *IL7R*, interleukin-12A (*Il12a*), and granzyme B (*Gzmb*) suggest the presence of natural killer cells and cytotoxic T cells (Fig. 1E) (*36*–*38*). Conversely, expression of the alarmin protein IL-33 was decreased in obese ferrets (*39*). We also noted reduced levels of surfactant protein B (*Sftpb*), a protein produced by epithelial cells involved in maintenance of surface tension in the lung (*40*), as well as decreased amphiregulin expression (*Areg*), suggesting impaired growth and proliferation of epithelial cells (Fig. 1F) (*41*, *42*). These data suggest that uninfected obese ferrets have impaired lung function and a pro-inflammatory environment even before infection, potentially altering viral pathogenesis in obese ferrets during influenza infection. However, further study and the continued development of addition ferret-specific reagents are needed to definitively make this conclusion.

### Influenza disease severity is increased in obese ferrets

**Fig. 2.**
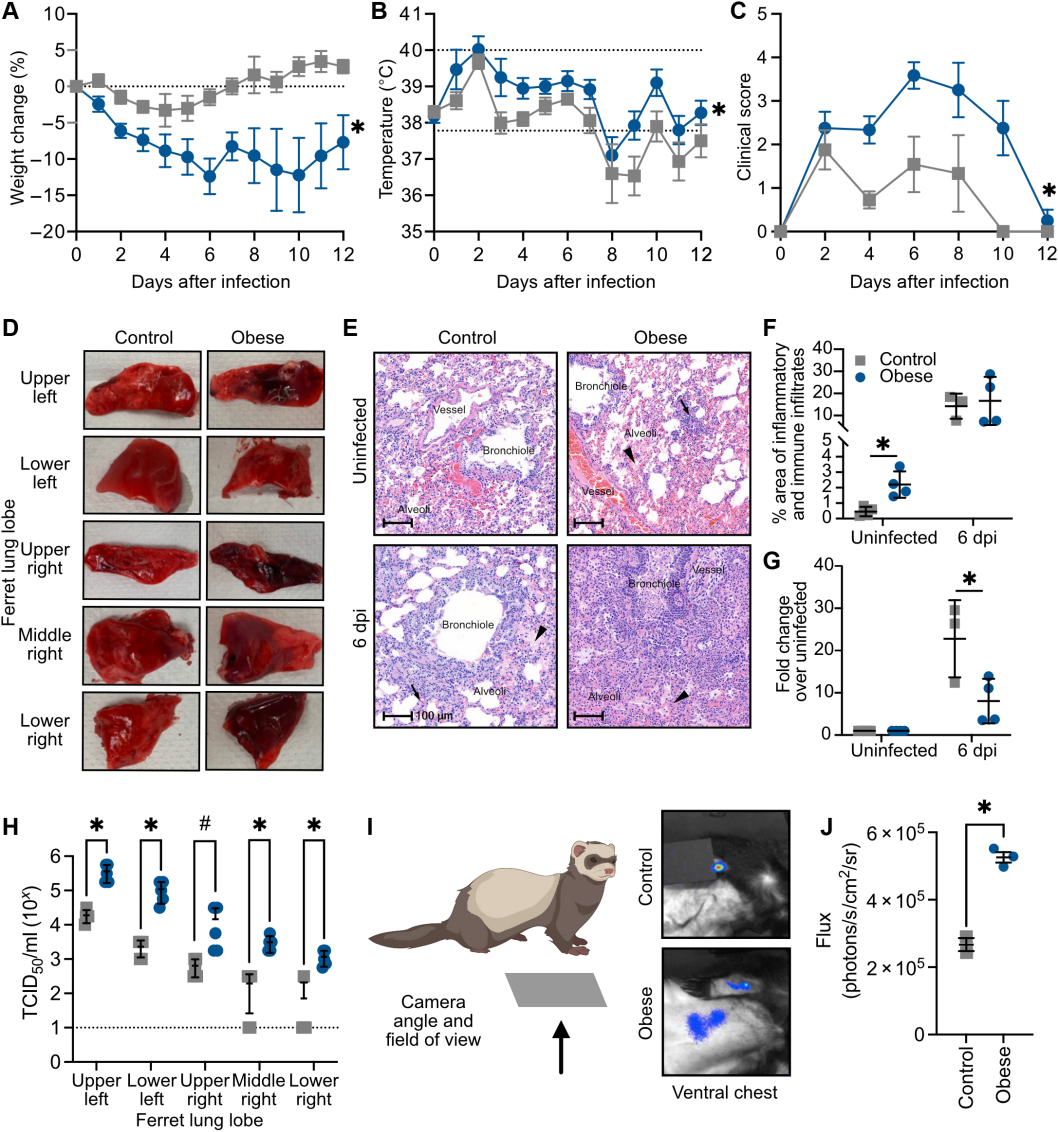
Disease severity following influenza infection is increased in obese ferrets. Male control and obese ferrets were inoculated with 10^6^ TCID_50_ A/California/04/2009 (H1N1) virus and monitored for 12 days. Daily (**A**) weight (*P* = 0.0002) and (**B**) temperature (*P* = 0.0032) were recorded. (**C**) Clinical scores (*P* = 0.0267) were recorded as described. Data represent two independent experiments of *n* = 4 to 6 per group. (**D**) Gross pathology of lungs from control and obese ferrets infected as in (A) at 6 dpi. Images represent *n* = 5 per group. (**E**) Hematoxylin and eosin (H&E) staining of lungs from uninfected control and obese ferrets, or ferrets infected as in (A) at 6 dpi. Data represent two independent experiments of *n* = 4 per group with six sections per ferret. Scale bars, 100 μm. (**F** and **G**) Quantification of total inflamed area as (F) percent of lung tissue (baseline, *P* = 0.0085) or (G) fold change over own uninfected (6 dpi, *P* = 0.0415). (**H**) Lungs were separated by lobe and TCID_50_ assays performed to measure viral titers at 3 dpi (upper left lobe, *P* = 0.0043; lower left, *P* = 0.0079; upper right, *P* = 0.0845; middle right, *P* = 0.0034; and lower right, *P* = 0.0031). Data represent two independent experiments with *n* = 2 per group. (**I**) Male control and obese ferrets were infected with 10^5.5^ TCID_50_ of A/California/04/2009-PA NLuc (H1N1) bioluminescent reporter virus. At 2 (*P* = 0.0175) dpi, the chest was imaged to detect virus in the lungs. Luminescence on the arm indicates substrate injection site. Illustration created with Biorender.com. (**J**) Quantification of (I). Data represent one independent experiment of *n* = 3 per group. Statistical significance was determined by mixed-effects analysis with repeated measures [(A) to (C), *P* value represents simple main effects analysis on diet], *t* test [(F), (G), and (J)], or multiple unpaired *t* tests (H). Error bars show SEM [(A) to (C) and (H)] or SD of the mean [(F), (G), and (I)]. Dashed lines indicate baseline weight (A), normal range (B), or lower limit of detection (H). *P < 0.05 and #*P* < 0.10.

The 2009 H1N1 influenza pandemic identified obesity as a risk factor for poor disease outcome (*43*, *44*). We hypothesized that like humans, obese ferrets would experience increased disease severity during influenza infection. To test, control and obese ferrets were inoculated with 10^6^ median tissue culture infectious dose (TCID_50_) of A/California/04/2009 (H1N1) virus, and we monitored the weight, body temperature, and clinical signs for 12 days postinfection (dpi). Obese ferrets lost significantly more weight and had higher body temperature during the acute phase of infection (Fig. 2, A and B, and fig. S3, A and B). We observed more disease symptoms in the obese ferrets, such as lethargy, coughing, and nasal discharge (Fig. 2C and fig. S3, C to I).

The increased clinical symptoms and disease severity led us to question whether there was greater progression to the lower respiratory tract than in the control group. We infected male ferrets with A/California/04/2009 (H1N1) virus and collected lungs at 6 dpi. We noted increased damage in the lungs of the obese ferrets by gross pathology (Fig. 2D). Histological analysis showed increased inflammation and immune infiltration in obese compared to control lungs at 6 dpi (Fig. 2, E to G), supporting an increase in overall disease severity in obese ferrets upon influenza virus infection. Influenza lesions of varying severity were observed in both the lean and obese infected groups in all sampled lung lobes. Lesions consisted of varying combinations of bronchitis, bronchiolitis, and alveolitis as well as peri-airway and perivascular mixed neutrophilic, lymphocytic, plasmacytic, and histiocytic inflammation (arrows). There was also alveolar edema and scattered foci of type II pneumocyte hyperplasia in more severely affected lobes of both groups. Uninfected obese animals had mild background inflammation and minor fluid accumulation in the alveolar spaces (Fig. 2, E to G). Viral titers were measured by TCID_50_ assay, treating the individual lobes of the lung as separate samples to spatially assess viral spread. Obese ferrets had overall significantly higher viral titers, even in the distal lung, suggesting that viral spread was increased (Fig. 2H) and/or faster viral clearance in the control group. To confirm more extensive viral spread at this time point, we infected control and obese ferrets with 10^5.5^ TCID_50_ of A/California/04/2009-PA NLuc, a bioluminescent reporter virus that allows longitudinal imaging of influenza virus (*45*, *46*). At 2 dpi, we detected significantly more luminescence in the obese ferrets including areas of the lower lung (Fig. 2, I and J).

Of note, there were minimal differences in disease symptoms in female ferrets. Obese female ferrets were not significantly larger than the control group, and there were no significant differences in markers of MetS (fig. S4, A to J). Disease severity was not significantly increased in obese female ferrets, although the body temperature and clinical scores trended higher in the obese group (fig. S4, K to M).

### Obesity alters the pulmonary response to influenza infection

**Fig. 3.**
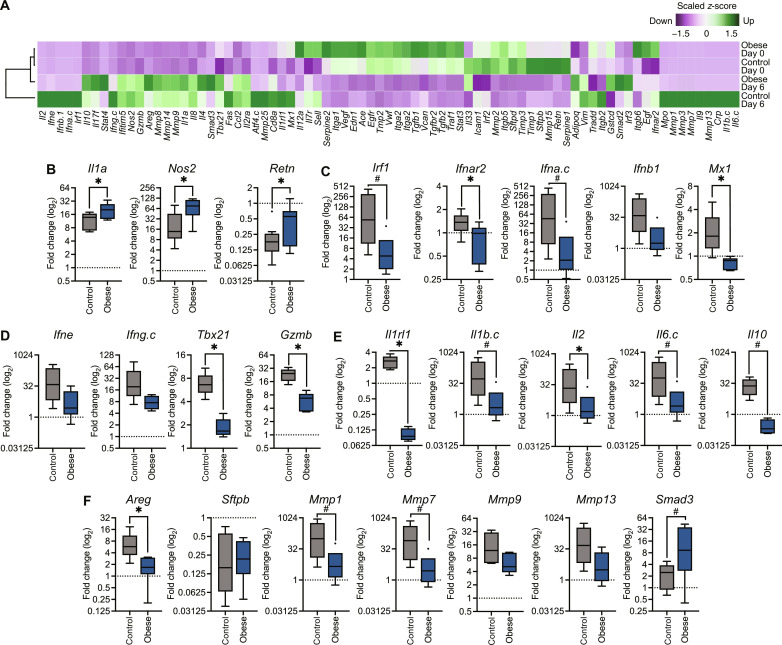
Altered inflammatory and immune gene expression in obese ferrets after infection. Male ferrets infected with 10^6^ TCID_50_ of A/California/04/2009 (H1N1) virus were euthanized at 6 dpi to collect lung tissue. Lungs were separated by lobe, and each lobe was treated as an individual sample. RNA was extracted from homogenates for high-throughput PCR. (**A**) Heatmap of gene expression normalized to uninfected control ferret using the 2^−ΔΔCt^ method. Obese infected ferrets group more closely with obese baseline (bars). Green indicates increased gene expression relative to uninfected control ferret, and purple indicates decreased. (**B** to **F**) Gene expression in obese and control ferret lungs after infection, normalized within diet groups to expression levels at baseline. (B) *Il1a*, *P* = 0.0342; *Nos2*, *P* = 0.0192; *Retn*, *P* = 0.0449. (C) *Irf1*, *P* = 0.0733; *Ifnar2*, *P* = 0.0218; *Ifna.c*, *P* = 0.0877; and *Mx1*, *P* = 0.0129. (D) *Tbx21*, *P* = 0.0001; and *Gzmb*, *P* < 0.0001. (E) *Il1rl1*, *P* = 0.0003; *Il1b.c*, *P* = 0.0934; *Il2*, *P* = 0.0440; *Il6.c*, *P* = 0.0953; and *Il10*, *P* = 0.0535. (F) *Areg*, *P* = 0.0110; *Mmp1*, *P* = 0.0833; *Mmp7*, *P* = 0.0890; and *Smad3*, *P* = 0.0513. Significance was determined by unpaired *t* test. Data represent the average of five lung lobes per *n* = 2 ferrets per group and one independent experiment of *n* = 2 per group. Samples were run in duplicate. Error bars represent minimum value to maximum value. *P < 0.05 and #*P* < 0.10.

Next, we assessed the pulmonary response to infection in the context of obesity (Fig. 3A). The gene expression profile at 6 dpi was chosen to assess lung repair and remodeling in addition to the transition from innate to adaptive immunity. In addition, obese ferrets still had high viral titers throughout the lungs at this time point. First, we compared gene expression in obese and control lungs after infection to control lungs at baseline to quantify global infection-related changes in gene expression. The gene profile of obese lungs at 6 dpi was more similar to uninfected lungs of both control and obese ferrets than to infected control lungs, indicating less of an overall change in antiviral state after infection in the obese ferrets (Fig. 3A). Next, we determined how the expression of individual genes was changing in infected control and obese ferrets compared to levels in diet-matched uninfected lungs. Notably, key inflammatory mediators, such as interleukin-1 (*Il1a*) and nitric oxide synthase 2 (*Nos2*) were up-regulated after infection in obese ferrets, possibly leading to extensive inflammatory damage of the respiratory epithelium as observed in histopathology (Fig. 3B) (*47*, *48*). Conversely, genes involved in the innate interferon (IFN) response were down-regulated in obese ferrets (Fig. 3C), as well as other antiviral defense mediators such as IFN-ε (*Ifne*), IFN-γ (*Ifng*), *Tbx21*, and *Gzmb* (Fig. 3D), suggesting dysregulation of adaptive immune cells (*49*, *50*). The antiviral mediator *Mx1* was lower in obese compared to that in control ferrets, mirroring results from both murine and human studies (Fig. 3C) (*51*, *52*). Although key players in the inflammatory response, interleukin-6 (*Il6.c*) and interleukin-1b (*Il1b*) also have protective effects after influenza infection (*53*, *54*) and show reduced up-regulation in obese lungs compared to control. This could be due to the heightened pro-inflammatory environment already present in obese lungs at baseline compared to that in control lungs and contribute the dysregulated antiviral response in obese ferrets after infection.

In addition to this reduced innate response, obese ferrets had lower IFN-γ (*Ifng*) and interleukin 2 (*Il2*) expression, suggesting dysregulation of adaptive immune cells (Fig. 3E) (*49*, *50*). Both interleukin-10 (*Il10*), a regulatory cytokine associated with decreased immunopathology during influenza infection (*55*), and interleukin-1 receptor like 1 (*Il1rl1*), the receptor for the alarmin IL-33 that alerts of epithelial trauma (*39*), were down-regulated in obese ferrets compared to control (Fig. 3E).

Last, we quantitated genes involved in lung function and repair (Fig. 3F). *Sftpb* levels were reduced in both control and obese ferrets, possibly because influenza infection results in the death of epithelial cells (*56*). However, control ferrets up-regulated *Areg* expression more than obese, suggesting an increase in epithelial cell regeneration (*41*, *42*), compared to obese. We also noted evidence of extracellular matrix remodeling in control ferrets as increased levels of matrix metalloproteases were detected after infection. These data suggest that obese ferrets may not adequately mount an immune response to infection and inefficiently repair lung tissue; however, further investigation throughout the course of infection is needed. Future tool development is needed to validate these findings at the protein level.

### Increased disease severity in obese ferrets is not strain-specific

**Fig. 4.**
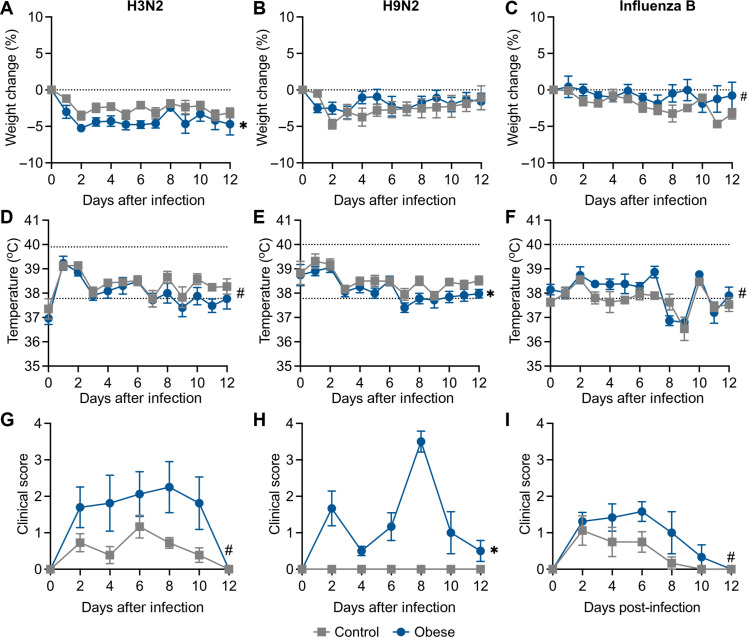
Increased disease severity in obese ferrets is independent of viral strain. Male control and obese ferrets were infected with 10^6^ TCID_50_ of A/Memphis/257/2019 (H3N2), A/Hong Kong/1073/1999 (H9N2), or B/Brisbane/60/2008 (influenza B) viruses and monitored for 12 days. (**A** to **C**) Weights (H3N2, *P* = 0.0165; H9N2, *P* = 0.3492; and influenza B, *P* = 0.0945), (**D** to **F**) body temperature (H3N2, *P* = 0.0772; H9N2, *P* = 0.0443; and influenza B, *P* = 0.0953), and (**G** to **I**) clinical scores (H3N2, *P* = 0.0614; H9N2, *P* = 0.0027; and influenza B, *p* = 0.0575) were recorded as described in Fig. 2. Data represent two independent experiments of *n* = 6 to 7 per group (H3N2), four independent experiments of *n* = 2 to 7 per group (H9N2), and two independent experiments of *n* = 3 to 5 per group (influenza B). Significance was determined by mixed-effects analysis with repeated measures (*P* value represents simple main effects analysis of diet). Error bars represent SEM, and dashed lines indicate baseline weight [(A) to (C)] or normal range [(D) to (F)]. *P < 0.05 and #*P* < 0.10.

Human obesity is more strongly associated with increased risk for some influenza strains versus others (*6*, *8*, *57*). To determine how obesity affects disease severity of different influenza virus strains, we chose representative viruses A/Memphis/257/2019 (human seasonal H3N2), A/Hong Kong/1073/1999 (avian-like H9N2), and B/Brisbane/60/2008 (influenza B, Victoria lineage) and challenged control and obese ferrets with 10^6^ TCID_50_ of H3N2 or H9N2 or with 10^5.5^ TCID_50_ of influenza B viruses. During H3N2 and influenza B infections, obese ferrets lost more weight. Yet, there were no observable differences with H9N2 infection (Fig. 4, A to C). Differences in body temperature were minimal (Fig. 4, D to F), but the obese group trended slightly higher at 3 to 5 dpi during influenza B infection (Fig. 4F). Differences in disease severity were predominantly symptomatic (Fig. 4, G to I, and figs. S5 to S7). Obese ferrets had significantly increased clinical scores compared to control, notably nasal discharge, cough, and excessive sneezing (figs. S5 to S7). H9N2 infection was particularly interesting in that it caused no discernable symptoms in control ferrets, yet obese ferrets had relatively high clinical scores. Further, we detected virus in the lungs of two H9N2-infected obese ferrets at 4 dpi; one in the lower left lobe (titer = 10^4.75^ TCID_50_/ml) and the other the lower right (titer = 10^4.75^ TCID_50_/ml). Histological analysis showed increased inflammation, immune cell infiltration, and overall damage in the obese lungs compared to control (fig. S6J). Together, obese ferrets were more likely to experience severe symptoms during influenza infection independent of strain (Table 1).

**Table 1.** Risk of symptoms by diet and influenza strain. H1N1, A/California/04/09; H3N2, A/Memphis/257/2019; H9N2, A/Hong Kong/1073/1999; Influenza B, B/Brisbane/60/2008.

Virus	Diet	Total ferrets	At-risk days	Symptomatic days	Average symptomatic days per ferret	Absolute risk	Relative risk for obese	*P* value
**H1N1**	Control	8	112	18	2.3	0.16	2.62 ± 1.65	<0.0001
	Obese	8	88	37	4.9	0.42		
**H3N2**	Control	16	184	48	3.2	0.26	2.27 ± 0.70	0.0001
	Obese	17	198	117	7.0	0.59		
**H9N2**	Control	13	146	15	1.1	0.10	1.91 ± 1.57	0.03
	Obese	13	122	24	1.7	0.20		
**Influenza B**	Control	8	68	16	1.9	0.24	1.75 ± 1.18	0.03
	Obese	8	68	28	3.5	0.41		
**Total**	Control	45	510	97	2.1	0.19	2.28 ± 0.52	<0.0001
	Obese	46	476	206	4.3	0.43		

### Obesity affects viral replication in the URT

**Fig. 5.**
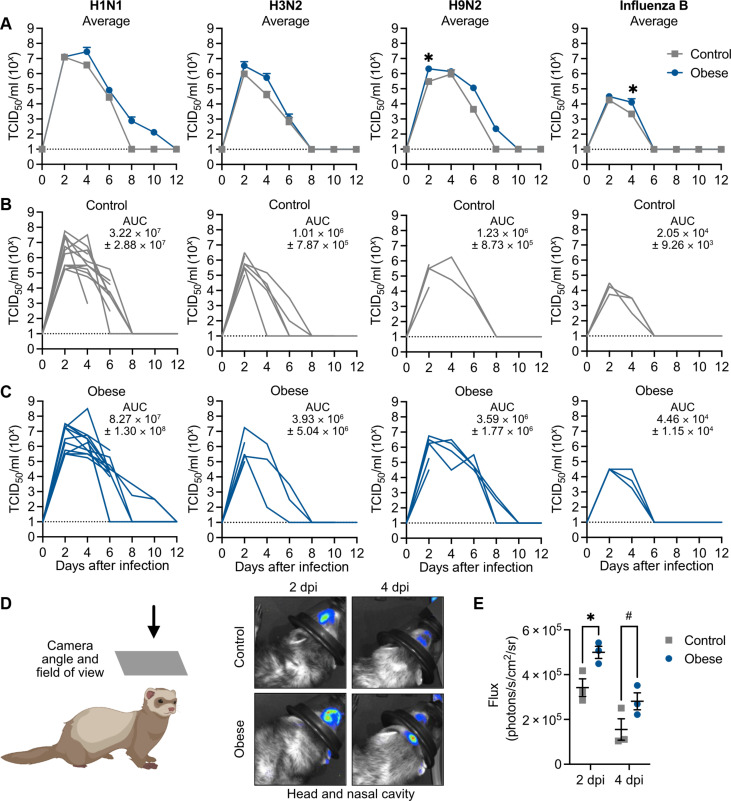
Increased viral replication in the URT of obese ferrets. (**A**) Male control and obese ferrets were infected with 10^6^ TCID_50_ of A/California/04/2009 (H1N1), A/Memphis/257/2019 (H3N2), and A/Hong Kong/1073/1999 (H9N2) or with 10^5.5^ TCID_50_ of B/Brisbane/60/2008 (influenza B) viruses. Nasal washes were collected every 48 hours and viral titers measured by TCID_50_ assay. H1N1, 4 dpi, *P* = 0.2793; H9N2, 2 dpi, *P* = 0.0275; and influenza B, 2 dpi, *P* = 0.0476, by two-way analysis of variance (ANOVA) with multiple comparisons. (**B**) Nasal wash titers of individual control ferrets and (**C**) obese ferrets shown in (A). Data represent two independent experiments of *n* = 4 to 7 per group. (**D**) Male control and obese ferrets were infected with 10^5.5^ TCID_50_ of A/California/04/2009-PA NLuc (H1N1) virus and the nasal cavity was imaged at 2 (*P* = 0.0412) and 4 (*P* = 0.0995) dpi. Illustration created with Biorender.com. (**E**) Quantification of (D). Statistical significance was determined by 2-way ANOVA with multiple comparisons. Data represent one independent experiment of *n* = 3 per group. Error bars show SEM (A) or SD of the mean (E), and dashed lines indicate the lower limit of detection. *P < 0.05 and #*P* < 0.10.

Because the upper respiratory tract (URT) is the primary site of infection, we next asked how obesity would affect viral replication in the nasal cavity by quantitating viral titers in nasal washes collected every 48 hours during the experiments shown in Figs. 2 and 4. Overall, obese ferrets trended toward increased viral titers in the URT and, in the case of H1N1 and H9N2 viruses, had detectable virus in the nasal washes for a longer duration (Fig. 5, A to C). Differences in viral load were most pronounced by 4 dpi except for H9N2 virus, where viral replication was increased as early as 2 dpi in the obese group. Viral replication at the peak of infection in the obese ferrets was higher during H1N1, H3N2, and H9N2 infection but not influenza B. We could visualize significant increases in luminescent flux in the nasal cavities of obese ferrets at 2 and 4 dpi using the bioluminescent reporter virus A/California/04/2009-PA NLuc (H1N1) as in Fig. 1, and signal in the obese ferrets persisted longer (Fig. 5, D and E).

### Obesity can affect viral transmission dynamics of a zoonotic virus

**Fig. 6.**
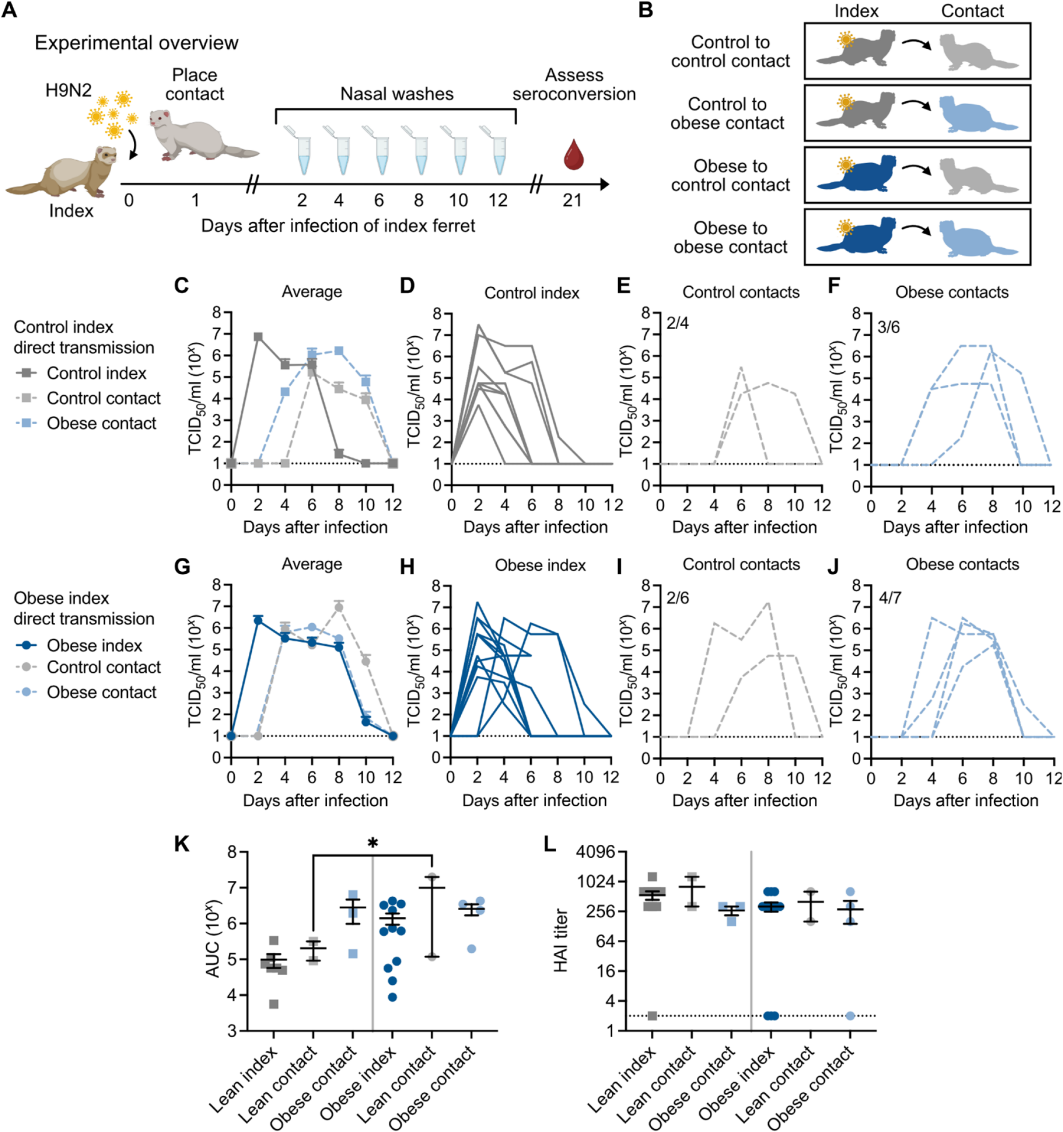
Obesity can affect viral transmission dynamics. (**A**) Experimental setup for a direct transmission study. An index ferret was inoculated with 10^6^ TCID_50_ A/Hong Kong/1073/1999 (H9N2) virus. After 24 hour, a naïve contact ferret was introduced. Nasal washes were collected every 48 hours for 12 days, and blood was collected at 21 dpi. (**B**) Schematic of the different transmission groups used in the study. Experiments were performed using male ferrets. Illustrations in (A) and (B) created with Biorender.com. (**C**) Nasal wash titers of control and obese ferrets paired with a control index ferret were measured by TCID_50_ assay. Average titer is shown. (**D**) Nasal wash titers of individual control index, (**E**) control contacts, and (**F**) obese contacts shown in (C). Number in top left indicates the number of ferrets that became influenza-positive/total number of contact ferrets. (**G**) Nasal wash titers of control and obese contacts paired with an obese index ferret. Average titer is shown. (**H**) Nasal wash titers of individual obese index, (**I**) control contacts, and (**J**) obese contacts paired with obese. (**K**) Area under the curve (AUC) of (B) and (E), control contacts *P* = 0.0270 by one way ANOVA with Sidak’s test. (**L**) Hemagglutination inhibition (HAI) assays were performed on plasma collected at 21 dpi. Control index versus obese contact paired with control *P* = 0.0793 by unpaired *t* test. Data represent three independent experiments of *n* = 3 to 4 transmission pairs per group. Error bars indicate SEM, and dashed lines indicate lower limit of detection in (C) to (J) and (L). *P < 0.05.

Because of the increased susceptibility of obese individuals to certain influenza strains (*6*) and the reduced antiviral response at 6 dpi observed in the obese ferrets (Fig. 3), we hypothesized that obese ferrets might be more susceptible to influenza infection. Because circulating human influenza strains transmit easily in the ferret model, we chose an avian-like virus, A/Hong Kong/1073/1999 (H9N2), with documented limited transmission ability between ferrets (*58*). Briefly, control ferrets were inoculated with 10^6^ TCID_50_ of H9N2 virus, and, 24 hours later, a control or obese naïve contact ferret was introduced into the same cage as an infected index ferret. Each pair was in direct contact and could interact freely. Nasal washes were collected from both the index and contact ferrets every 48 hours to monitor viral transmission, and, at 21 dpi, blood was collected to assess seroconversion. We included both homologous and heterologous diet pairings (Fig. 6, A and B).

When paired with control index ferrets, obese contacts retained the phenotype of higher viral titers and increased shed duration compared to control contacts (Fig. 6, C to F, and Table 2). Regardless of the diet status of the contact ferret, the transmission rate of H9N2 virus from a control index ferret was 50%; however, obese contacts became infected earlier and shed virus longer than control contacts (Fig. 6, E and F, and Table 2).

**Table 2.** H9N2 transmission dynamics. dpi, days postinfection; HAI, hemagglutination inhibition.

Group	Contact	Time to infection (dpi)	Time of peak viral titer (dpi)	Peak viral titer (10^*x*^ TCID_50_/ml)	Day of viral clearance (dpi)	Duration of viral shed (days)	HAI titer
**Control to control**	Contact 1	6	6	5.50	8	2	320
	Contact 2	6	8	4.75	12	6	1280
	Contact 3	–	–	–	–	–	–
	Contact 4	–	–	–	–	–	–
**Control to obese**	Contact 1	6	8	6.25	12	6	320
	Contact 2	4	6	4.75	10	6	320
	Contact 3	4	6	6.50	10	6	160
	Contact 4	–	–	–	–	–	–
	Contact 5	–	–	–	–	–	–
	Contact 6	–	–	–	–	–	–
**Obese to control**	Contact 1	6	8	4.75	12	6	160
	Contact 2	4	8	7.30	10	6	640
	Contact 3	–	–	–	–	–	–
	Contact 4	–	–	–	–	–	–
	Contact 5	–	–	–	–	–	–
	Contact 6	–	–	–	–	–	–
**Obese to obese**	Contact 1	6	8	5.25	10	4	160
	Contact 2	6	6	6.50	10	4	320
	Contact 3	4	4	6.50	10	6	640
	Contact 4	4	6	6.25	12	8	320
	Contact 5	–	–	–	–	–	–
	Contact 6	–	–	–	–	–	–
	Contact 7	–	–	–	–	–	–

We next considered the possibility that obese ferrets may be more likely to transmit, as increased viral shed can facilitate transmission (*59*). We repeated the experiment using obese ferrets as indices. Again, obese contacts retained the phenotype of increased viral titers and duration of shed (Fig. 6, G to J, and Table 2). Obese ferrets transmitted to only two of the six control contacts (33%; Fig. 6I), with only one control contact displaying robust viral shed. Conversely, obese ferrets successfully transmitted to four of the seven obese contacts (57%; Fig. 6J). We concluded that while transmission efficacy did not appear to be altered on the basis of the diet of the contact ferret, the obese ferrets may be more susceptible to infection because two of the three obese contacts became positive for influenza virus earlier than control contacts (Fig. 6F and Table 2). The viral titers of obese ferrets had similar area under the curve (AUC) regardless of index diet. While we detected active viral replication in only two control contact ferrets, making statistical comparisons difficult due to low numbers and high variation, we did note greater AUC in control ferrets paired with obese index compared to those paired with control (Fig. 6K). Intriguingly, obese contacts shed greater quantities for a longer period than control index ferrets, while no difference in AUC was noted in obese index pairings (Fig. 6K). Last, when assessing seroconversion by hemagglutination inhibition (HAI) assay, obese contacts paired with control ferrets had decreased HAI titers compared to control contacts paired with control index. Further, control contacts paired with obese index trended lower that control contacts paired with control (Fig. 6L and Table 2). Together, obesity may influence transmission dynamics of H9N2 virus.

## DISCUSSION

In 2022, approximately 800 million adults, children, and adolescents were considered obese (BMI > 30). In some countries, more than 40% of the population are obese with levels continuing to rise. This is concerning given the health risks associated with a high BMI including increased risk of developing severe disease with respiratory viruses like SARS-CoV-2 and influenza. In these studies, we developed an obese ferret model to study the impact of obesity on disease severity and transmission of influenza virus. Like humans (*6*), obese ferrets experienced increased disease severity during H1N1 infection as shown by increased weight loss and body temperature, as well as greater severity and duration of symptoms. Viral spread and lung injury were increased in obese ferrets, which is similar to humans where case reports of bronchiolitis, alveolar hemorrhage, and increased virus in the lungs of obese decedents has been reported (*60*). As ferrets are known to recapitulate the human respiratory tract, our model helps translate these discoveries to the clinic. While rodent models have provided valuable insight into mechanisms of disease severity in the context of obesity (*14*, *61*), the obese ferret and associated reagents provide a robust and translatable model organism for vaccine and antiviral testing and for risk assessments in surveillance and epidemic response scenarios.

In our model, we used influenza strains displaying a spectrum of symptom severity from mild to severe. For each virus that we tested, the obese group was more likely to experience more severe and prolonged symptoms compared to control (Table 1). Unexpectedly, H9N2, a virus typically not detected in the lungs of ferrets (*62*), spread to the lower respiratory tract of two of the obese ferrets yet caused no observable clinical symptoms in control ferrets (Fig. 4). In addition, obese ferrets had higher viral titers in the nasal wash, congruent with data suggesting obese individuals experience increased viral shedding for longer periods of time during both H1N1 and H3N2 infection compared to non-obese, even when asymptomatic (*7*). In our hands, the obese ferrets experienced prolonged viral shed in the case of H1N1 and H9N2 infections but cleared virus on the same day as control ferrets during H3N2 and influenza B infections. Human cohort studies also report strain-specific phenomena; one study found obesity had no bearing on human symptom severity during seasonal H3N2 infection as opposed to H1N1 (*6*), and obese ferrets had lower symptom scores during H3N2 infection compared to H1N1.

The increased viral spread to the lower respiratory tract in obese ferrets could be explained by the dampened antiviral response in the lungs of obese ferrets. Blunted type I IFN responses are described in both mouse models and human epithelial cells in vitro (*17*, *18*). Further, obese ferrets did not adequately transcriptionally up-regulate amphiregulin, matrix metalloproteinases, or the alarmin-activating receptor IL-1RLI, genes involved in epithelial regeneration and wound healing. Obese ferrets also had lower levels of surfactant protein B and amphiregulin at baseline, which could compromise tensile strength of the lung and maintenance and turnover of the pulmonary epithelium, leaving the lungs in an inherently weakened state. In addition, homeostasis in the obese ferrets was characterized by high levels of inflammatory cytokines and proapoptotic protein FAS, which could compromise lung function.

Obesity also affected viral transmission of an avian-like influenza virus. Obese ferrets were more susceptible to infection through direct contact independently of the index ferret’s diet. Although the rate of transmission from a control index was the same whether the contact was control or obese (50%), the obese ferrets were likely to become infected earlier. Unusually, an avian influenza virus transmitted more efficiently from obese index ferrets to obese contacts (57%), suggesting that obesity affects both transmission efficiency and susceptibility in the ferret model. Despite the lower likelihood of transmission from an obese to a control ferret (33%), one of the two control contacts that became infected shed virus earlier, had higher viral loads, and prolonged shed similar to our findings in obese ferrets. Honce *et al.* (*18*) reported that influenza viruses became more virulent as they were passed through an obese host but not control, suggesting that, in some cases, virus shed by the obese ferrets may cause more severe infection. Using the more translatable ferret model to characterize the impact of obesity on viral evolution and bottlenecks in transmission dynamics is warranted as the rate of obesity continues to rise and may be valuable as a tool in risk assessment of emerging influenza viral strains.

Increased disease severity during H1N1 infection could not be replicated using female ferrets. Metabolism can be sex-dependent (*63*), and this may explain the less marked difference in disease severity in females; however, all ferrets used in this study were neutered or ovariectomized. We did note variation among individual ferrets, particularly in HDL levels (fig. S1), and we were unable to perform truly fasted measurements due to the complex dietary needs of ferrets, which could lead to variation. HDL levels can also vary in humans due to factors such as underlying health conditions and environmental factors (*64*, *65*). In humans, different hallmarks of MetS are also more likely to occur in males versus females (*66*). Metabolic dysfunction can also occur in the absence of phenotypic adiposity, leading to consequences such as diabetes and cardiovascular disease; conversely, individuals with larger BMIs can be metabolically healthy (*67*, *68*). In female ferrets, our obese diet protocol did not significantly increase ferret body mass or alter biochemical hallmarks of metabolic dysfunction, suggesting that a different or prolonged approach may be necessary to induce ferret obesity and recapitulate increased disease severity in female ferrets. Further, applying our model to intact ferrets, or in other high-risk presentations such as pregnancy or age status, could shed light on the interactions among sex hormones, metabolic dysfunction, and immune responses on influenza disease outcomes [reviewed in (*69*)].

With every study, any caveats and limitations must be considered. Our study and those involving human cohorts were conducted with different methodology. For example, we defined viral shed by the detection of infectious virus, while, in human studies, the presence of viral genome is typically detected by the more sensitive quantitative polymerase chain reaction (qPCR) assay (*6*, *7*, *59*). Further, the definition of symptom severity in clinical studies varies and is often self-reported data that include criteria non-applicable to ferret studies, such as hospitalization, intensive care unit admission, and death. Observational scoring is also a subjective metric, so we used multiple persons to independently score our ferrets. Although we assessed gene expression in the ferret lung, due to the paucity of ferret reagents, we were unable to validate these findings at the protein level limiting our statistical understanding of gene ontology. Optimization of our primers is ongoing as improved genome builds become public, which will also enable more robust bioinformatic analysis of gene ontology at baseline and after infection. Last, ferret studies are typically low *n* due to complex husbandry needs, limiting our statistical power (*12*). However, our studies align with and further a large body of research spanning work in cell culture, mouse models, immunology, and epidemiology and point to a conserved effect of metabolic dysfunction on the viral infection dynamics.

Here, we show that ferrets fed with calorically dense diets recapitulate features of obesity and metabolic dysfunction seen in humans and display increased influenza-induced disease severity. The ferret reagents created for this study help us unravel the mechanisms of increased viral pathogenesis associated with obesity, including reduced antiviral responses and impaired wound healing, and will be key for studies beyond investigating the impact of diet on infectious disease. We report obesity influences both transmission dynamics and infection kinetics, potentially due to the increased viral replication in the URT and the prolonged duration of viral shed. Beyond obesity, future studies using our model could be used to determine what aspects of MetS most affect influenza and other disease outcomes. Further, this model will be invaluable for testing next-generation vaccine and antiviral designs, as these pharmaceutical interventions traditionally show poor protection in durability in obese populations (*70*, *71*). These results, together with the increasing availability of ferret reagents (*72*, *73*), can help us understand many aspects of influenza disease in the context of obesity and beyond, such as immunological responses, risk assessment, and refinement of vaccines, antivirals, and other therapeutics to help protect this and other high-risk populations.

## MATERIALS AND METHODS

### Experimental design

The objective of the study was to determine mechanisms of disease severity in the context of obesity using the gold-standard animal model for influenza studies. Before study, ferret diets were optimized with the supervision of veterinarians in the St. Jude Children’s Research Hospital Animal Resource Center, and primers targeting genes of interest were stringently validated.

### Ethics statement

All animal experiments (protocol no. 513) were approved by the St. Jude Children’s Research Hospital Institutional Animal Care and Use Committee. Ferrets were housed at ambient temperature (20°C and 45% relative humidity) with 12-hour light cycles and provided species and study appropriate enrichment activities.

### Ferret diets and measurements

Neutered, de-scented male ferrets or ovariectomized female ferrets were obtained from Triple F Farms (Elmyra, NY) at the age of 6 weeks and randomly assigned to obese or control diet groups. Diets were allocated daily for 12 weeks (table S1). All ferrets were provided water ad libitum. Control ferrets were provided a restricted volume of high-density ferret chow (Lab Supply 5LI4; 17% carbohydrates, 36% protein, and 47% fat) once daily. Obese ferrets received a 1:1 mix of high-density ferret diet and feline diet (Lab Supply 5003; 40% carbohydrates, 32% protein, and 27% fat) ad libitum and supplemented with wet kitten food (Iams 131321, Chewy.com) once per day beginning at 9 weeks of age. Throughout the diet period, physical measurements were recorded weekly. Ferret body length was measured from nose to base of tail and waist circumference measures just above the iliac crest. Skinfold fat was determined using a digital fat caliper on the right side of the abdomen above the iliac crest. Ferret mass index was calculated by standardizing the product of weight in kilograms and circumference in centimeters by the ferret length in squared centimeters

$$\text{Ferret mass index}\left(\text{FMI}\right)=\frac{\text{weight}\left(\text{kg}\right)\times \text{circumference}\;\left(\text{cm}\right)}{\text{length}{\left(\text{cm}\right)}^{2}}$$ (1)

### Blood chemistry analyses

Ferrets were sedated with 4% isoflurane, and blood was collected via the jugular vein into a heparinized collection tube (Becton Dickinson, 366643). Plasma was separated via centrifugation and stored at −80°C until use. Blood chemistry analyses were performed by the Veterinary Pathology Core at St. Jude Children’s Research Hospital. Leptin levels were determined using Human Leptin Instant ELISA kit (Invitrogen BMS2039INST) according to the manufacturer’s instructions.

### Viruses and cells

Madin-Darby canine kidney (MDCK) cells (American Type Culture Collection, CCL-34) were cultured in minimal essential medium (MEM) (Corning, 10-010-CV) supplemented with 10% fetal bovine serum (HyClone) and 200 mM l-glutamine (Gibco, 35050079). Cells were incubated at 37°C/5% CO_2_.

To propagate A/California/04/2009 (H1N1), A/California/04/2009-PA NLuc (H1N1), A/Memphis/257/2019 (H3N2), and B/Brisbane/60/2008 (Victoria lineage) viruses, MDCK cells were inoculated with virus diluted in infection media [MEM supplemented with 200 mM l-glutamine, 0.075% bovine serum albumin (Gibco, 15260-037), and *N*-tosyl-l-phenylalanine chloromethyl ketone (1 μg/ml)–treated trypsin (Worthington, LS003740)] for 72 hours at 37°C/5% CO_2_. Supernatants were harvested and clarified by centrifugation, and aliquots were stored at −80°C until use. A/Hong Kong/1073/1999 (H9N2) virus was propagated in 9-day-old embryonated chicken eggs as previously described (*74*). Viral titers were measured by TCID_50_. Briefly, MDCK cells were seeded in 96-well plates at a density of 30,000 per well and inoculated in triplicate with tenfold serial dilutions of samples or viral stock diluted in infection media. Cells were incubated for 72 hours at 37°C/5% CO_2_. Viral titers were read by hemagglutination of 0.5% packed turkey red blood cells and calculated by the method of Reed and Muench (*75*).

### Infections

Ferrets were lightly sedated with 4% inhaled isoflurane and inoculated with virus diluted in phosphate-buffered saline (PBS) (Corning, 21-040-CV) supplemented with penicillin (100 U/ml) and streptomycin (100 μg/ml; Corning 30-002 CI). The dose of virus given was as follows: 10^6^ TCID_50_ of A/California/04/2009 (H1N1), A/Memphis/257/2019 (H3N2), and A/Hong Kong/1073/1999 (H9N2); 10^5.5^ TCID_50_ of A/California/04/2009-PA NLuc (H1N1) and B/Brisbane/60/2008 (influenza B, Victoria lineage). Weight and temperature were assessed daily. Clinical scores were assessed by observing the presence and severity of symptoms using a point system. Metrics included sneezing (none = 0, mild = 1, and excessive = 2), coughing (absence = 0 and presence = 1), nasal discharge (absence = 0 and presence = 1), conjunctivitis (absence = 0 and presence = 1), and lethargy (active and playful = 0, active when stimulated = 1, and not active when stimulated = 2). Mild sneezing was defined as one to two sneezes during the observation period, while continuous sneezing was considered excessive. Clinical scores were assessed by at least two investigators. Nasal washes were graded as follows: clear = 0, cloudy = 1, mucus present with discoloration = 2, and thickened mucus present = 3. Data are presented as the sum of points.

“At-risk days” refers to total days of observation summed across all ferrets studied. “Symptomatic days” refers to days with observable symptoms (a symptom score of >0). “Average symptomatic days per ferret” is symptomatic days divided by total ferrets. “Absolute risk” is calculated as the total days with symptoms divided by at risk days (Eq. 2a). “Relative risk” is calculated as the absolute risk of obese divided by the absolute risk of control ferrets (Eq. 2b) with error defined as plus or minus the 95% confidence interval (Eq. 2, c and d)

$$\text{Absolute risk}\left(\text{AR}\right)=\frac{\text{Total symptomatic days}}{\text{At}-\text{risk days}}$$ (2a)

$$\text{Relative risk}\left(\text{RR}\right)=\frac{\text{AR}\left(\text{obese}\right)}{\text{AR}\left(\text{control}\right)}=\frac{\frac{\text{Total symptomatic days}\left(\text{obese}\right)}{\text{At}-\text{risk days}\left(\text{obese}\right)}}{\frac{\text{Total symptomatic days}\left(\text{control}\right)}{\text{At}-\text{risk days}\left(\text{control}\right)}}$$ (2b)

$$\begin{array}{c} \text{Standard error of relative risk}\left(\text{SE}\right)=\\ \sqrt{\frac{1}{\text{Total symptomatic days}\left(\text{obese}\right)}+\frac{1}{\text{Total symptomatic days}\left(\text{control}\right)}+\frac{1}{\text{At}-\text{risk days}\left(\text{obese}\right)}+\frac{1}{\text{At}-\text{risk days}\left(\text{control}\right)}} \end{array}$$ (2c)

$$95\%\text{CI}=e^{\text{ln}\left(\text{RR}\right)-1.96\times \text{SE}}\;\text{to}\;e^{\text{ln}\left(\text{RR}\right)+1.96\times \text{SE}}$$ (2d)

### Tissue collection

Ferrets were humanely euthanized via cardiac injection of Euthasol (Patterson Veterinary Supply). Animals euthanized after 12 weeks on diet were necropsied. Abdominal adipose tissue was removed and weighed. For infection studies, individual lobes of the lungs were resected carefully to avoid cross-contamination. Lung tissues were finely minced with scissors before adding 1 ml of sterile PBS and bead beaten. Samples were centrifuged for 5 min at 1500*g* to clarify before measuring viral titers by TCID_50_ assay. For histological analysis, whole tissues were submerged in 10% neutral buffered formalin for at least 48 hours. After fixation, tissues were embedded in paraffin, sectioned at 4 μm, mounted on positively charged glass slides (Superfrost Plus, Thermo Fisher Scientific), and dried at 60°C for 20 min before dewaxing and staining with hematoxylin and eosin (H&E) (Richard-Allan Scientific). Coverslips were placed using the HistoCore SPECTRA Workstation (Lecia Biosystems). Pathology evaluation was conducted blindly to the experimental conditions. H&E-stained sections were scanned to a ×20 scalable magnification using a PANNORAMIC 250 PIII slide scanner (3DHistech). The extent of immune infiltration in the different experimental conditions was evaluated visually and then quantified on whole slide images using HALO v3.5.4134.137 and HALO AI 3.6.4134 (Indica Labs).

### Bioluminescent imaging

Ferrets infected intranasally with A/California/04/2009-PA NLuc virus were imaged as previously described (*45*). Briefly, ferrets were anesthetized with 4% inhaled isoflurane, and the chest area directly above the lung was shaved to minimize background. Ferrets were injected via the cephalic vein with Nano-Glo substrate (Promega N1110) at a dilution of 1:5 in sterile PBS. Ferrets were imaged using a Xenogen IVIS200 (PerkinElmer) with an exposure time of 5 min. Data were analyzed with LivingImage software (PerkinElmer).

### Nasal wash collection

Ferrets were anesthetized intramuscularly with ketamine (30 mg/kg; Patterson Veterinary Supply), and sneezing was induced by adding 1 ml of PBS supplemented with penicillin (100 U/ml) and streptomycin (100 μg/ml) dropwise to the nares. Sample was collected into a sterile specimen cup, briefly centrifuged, and stored at −80°C until use. Viral titers were determined via TCID_50_ assay as described.

### High-throughput qPCR and primer validation

Primers targeting immune and lung function-related genes of interest were designed on the basis of the ferret genome sequences provided by Ensembl (https://useast.ensembl.org/Mustela_putorius_furo/Info/Index). Primer validation was conducted as described by the manufacturer (*76*). Tissues were collected from multiple ferrets, and RNA was extracted using the RNeasy RNA extraction kit (QIAGEN, 74104) including an on-column deoxyribonuclease (DNase) digestion using the ribonuclease-free DNase set (QIAGEN, 79254). RNA was quality-assessed and quantified using a NanoDrop 8000 spectrophotometer. cDNA was synthesized with the SuperScript IV VILO master mix (Invitrogen 11756050) per the manufacturer’s instructions. Specific target amplification was performed with the Preamplification Master Mix (Fluidigm, PN 100-5580) and candidate primers targeting genes of interest (table S2) at 95°C for 10 min, followed by 12 cycles of 95°C for 15 s and 60°C for 4 min followed by exonuclease I (New England Biolabs, PN M0293S) treatment at 37°C for 30 min and 80°C for 15 min to remove any unincorporated primers. The resulting samples were diluted 1:5 using DNA suspension buffer (Teknova, T0221). These samples were considered the sample at the highest concentration. Using DNA suspension buffer, we made 15-step twofold dilution series in DNA suspension buffer. Samples were prepared with SsoFast EvaGreen Supermix (Bio-Rad, PN 172-5211), loaded onto a primed 96.96 internal fluidics chip (IFC) (Fluidigm BMK-96.96), and run in triplicate against each individual primer to be validated along with at least three non-template controls. Data were analyzed using a Fluidigm Biomark HD and Fluidigm Real-Time PCR Analysis version 4.7.1. Threshold cycle (Ct) values that fell in the range of 5 to 24 for each combination of assays were accepted. ΔCt values for each dilution step from the previous dilution samples were calculated and used to plot the mean slope of ΔΔCt against template concentration. The ideal slope would be 0, and we considered validated assay to fall in the range of −0.1 to 0.1. To further validate, PCR product was run on an agarose gel, bands were extracted and and sequenced via the Sanger method to ensure that the correct gene was amplified. After validation, experimental samples were prepared using the above protocol; however, the preamplification step was done using pooled primers. Experimental samples were run in duplicate and analyzed via the −ΔΔCt method. Data were normalized to expression at either baseline within diet groups or baseline of control, uninfected lungs for graphical display of individual gene expression data. Gene ontology was performed using the Database for Annotation, Visualization and Integrated Discovery. Relative up- and down-regulated gene sets were first uploaded and then compared to the background set of all tested genes in the *Homo sapiens* background with the top functional groups reported. For relative gene expression visualization using R function heatmap, gene expression was normalized to endogenous actin levels and scaled using the scale function, and heatmap was clustered on the basis of expression patterns in both samples and genes (*77*, *78*).

### Transmission experiments

Singly housed ferrets were lightly sedated with 4% inhaled isoflurane and inoculated with indicated virus diluted in 500 μl of PBS. Directly inoculated ferrets are referred to as index ferrets. After 24 hours, a naïve ferret was placed in the same cage with the index ferret. Ferrets could interact freely. For transmission between unlike diet groups, diets remained fixed for the index ferret. Weight, body temperature, and clinical signs were monitored as before. Nasal washes were collected every 48 hours to monitor viral transmission.

### HAI assay

Blood was collected as described. Samples were centrifuged at 5000*g* for 15 min to separate the plasma and stored at −80°C until use. The red blood cell pellet was discarded. Samples were incubated with receptor-destroying enzyme (RDE; Denka Seiken, Tokyo) overnight at 37°C. RDE-treated samples were inactivated at 56°C for 1 hour. PBS was added to a final dilution of 1:10, and samples were stored at −80°C for at least 4 hours to ensure complete inactivation of any residual neuraminidase. Twofold sample dilutions were added to a 96-well plate and incubated with 4 hemagglutinating units of the indicated virus for 15 min at room temperature. Packed turkey red blood cells (0.5%) diluted in PBS were added to each well, and plates were incubated at 4°C for 90 min. HAI titer is reported as the reciprocal dilution of the last positive well. Positive and negative controls, as well as back titrations of virus, were included on each individual plate. If back titration value was >1:8, then the assay was repeated.

### Statistical analysis

Data were analyzed using GraphPad Prism version 9.5.1 using the appropriate statistical test as indicated in the figure legends. Final figures were prepared using Affinity Designer (Serif).
