# Supplementary material for: Diet-induced obesity affects influenza disease severity and transmission dynamics in ferrets
Source: Sci Adv. 2024 May 10;10(19):eadk9137. doi: 10.1126/sciadv.adk9137 (PMC11086619; doi:10.1126/sciadv.adk9137)
Supplement: Supplementary file 1 — Figs. S1 to S7 Tables S1 and S2 [file sciadv.adk9137_sm.pdf]

Science Advances
AAAS

Supplementary Materials for

Diet-induced obesity affects influenza disease severity and transmission dynamics in ferrets

Victoria Meliopoulos *et al.*

Corresponding author: Stacey Schultz-Cherry, stacey.schultz-cherry@stjude.org

Sci. Adv. **10**, eadk9137 (2024)
DOI: 10.1126/sciadv.adk9137

This PDF file includes:

Figs. S1 to S7
Tables S1 and S2

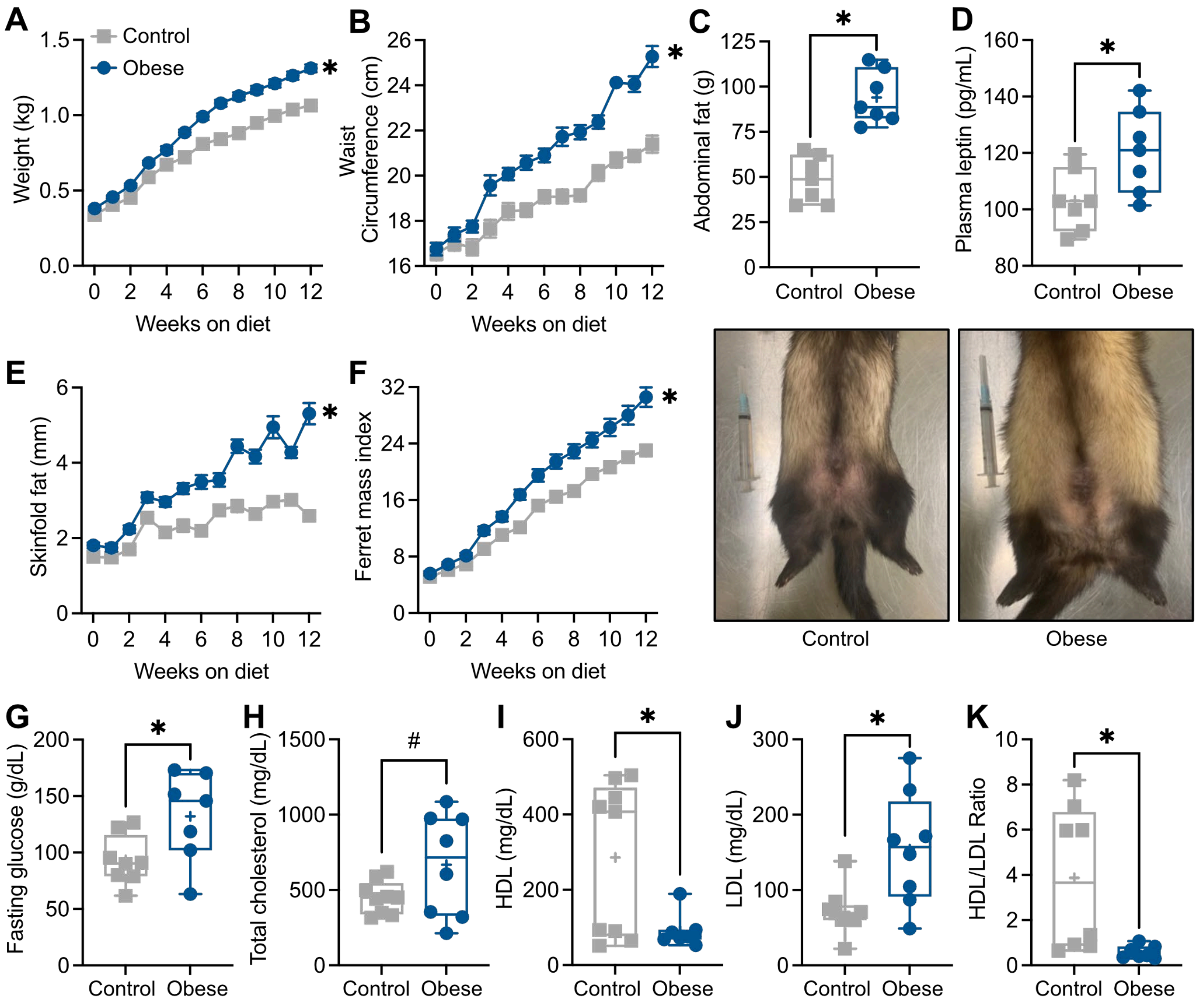

Fig. S1. Inducing obesity and metabolic syndrome in ferrets. Male ferrets (6 weeks of age) were placed on control or high calorie diet and monitored for 12 weeks. (A) Weight (p<0.0001), (B) waist circumference (p<0.0001), and (C) skinfold fat (p<0.0001) were assessed weekly. (D) Ferret mass index was calculated from physical measurements (p<0.0001). Data was analyzed by 2-way ANOVA with repeated measures (p-value represents simple main effect of diet). (E) Animals were sacrificed post-diet and abdominal fat deposits removed and weighed (p<0.0001 by unpaired t test). (F) Plasma leptin levels post-diet were determined by ELISA (p=0.0278 by unpaired t test). (G) Differences in physical appearance of control and obese ferrets. (H) Fasting glucose (p=0.0309), (I) total cholesterol (p=0.0878), (J) high-density lipoprotein (HDL) (p=0.0265), and (K) low-density lipoprotein (LDL) (p= 0.0079) levels in the plasma of ferrets post-diet. (L) Ratio of HDL to LDL (p=0.0187). Significance of (H-L) was determined by unpaired t test. Data represents 2 independent experiments of n=7-10/group. Error bars represent standard error of the mean (SEM) (A-D) or minimum value to maximum value (E-F, H-L). *p<0.05, $^{\#}p$<0.10.

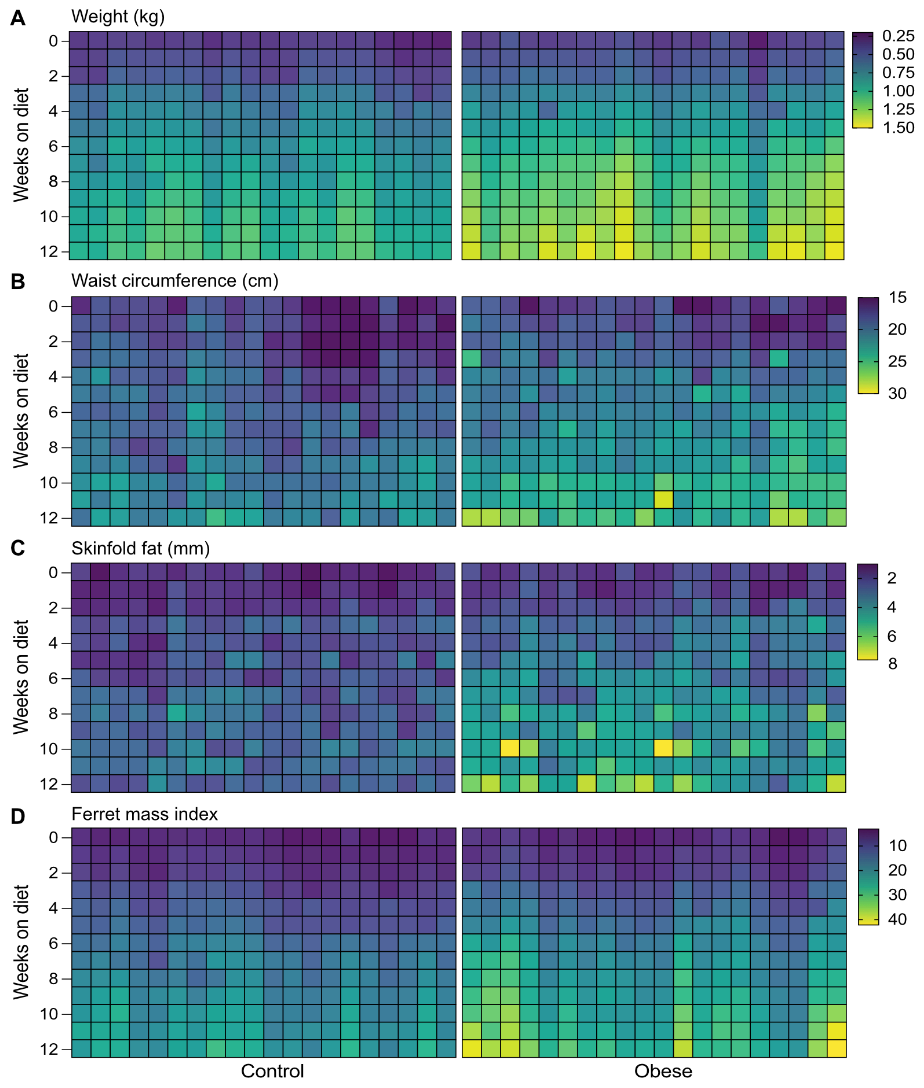

Fig. S2. Individual growth kinetics of ferrets on diet protocol. Male ferrets (n=40 total) placed on diet protocol with individual ferret growth recorded as (A) weight measured in kilograms, (B) waist circumference measured in centimeters, (C) skinfold fat measured in millimeters, and (D) ferret mass index measured by standardizing the product of weight in kilograms and circumference in centimeters by the ferret length in centimeters squared. Each column is representative of one ferret measured weekly for 12 weeks. Individual data underlies average values plotted in Fig. S1.

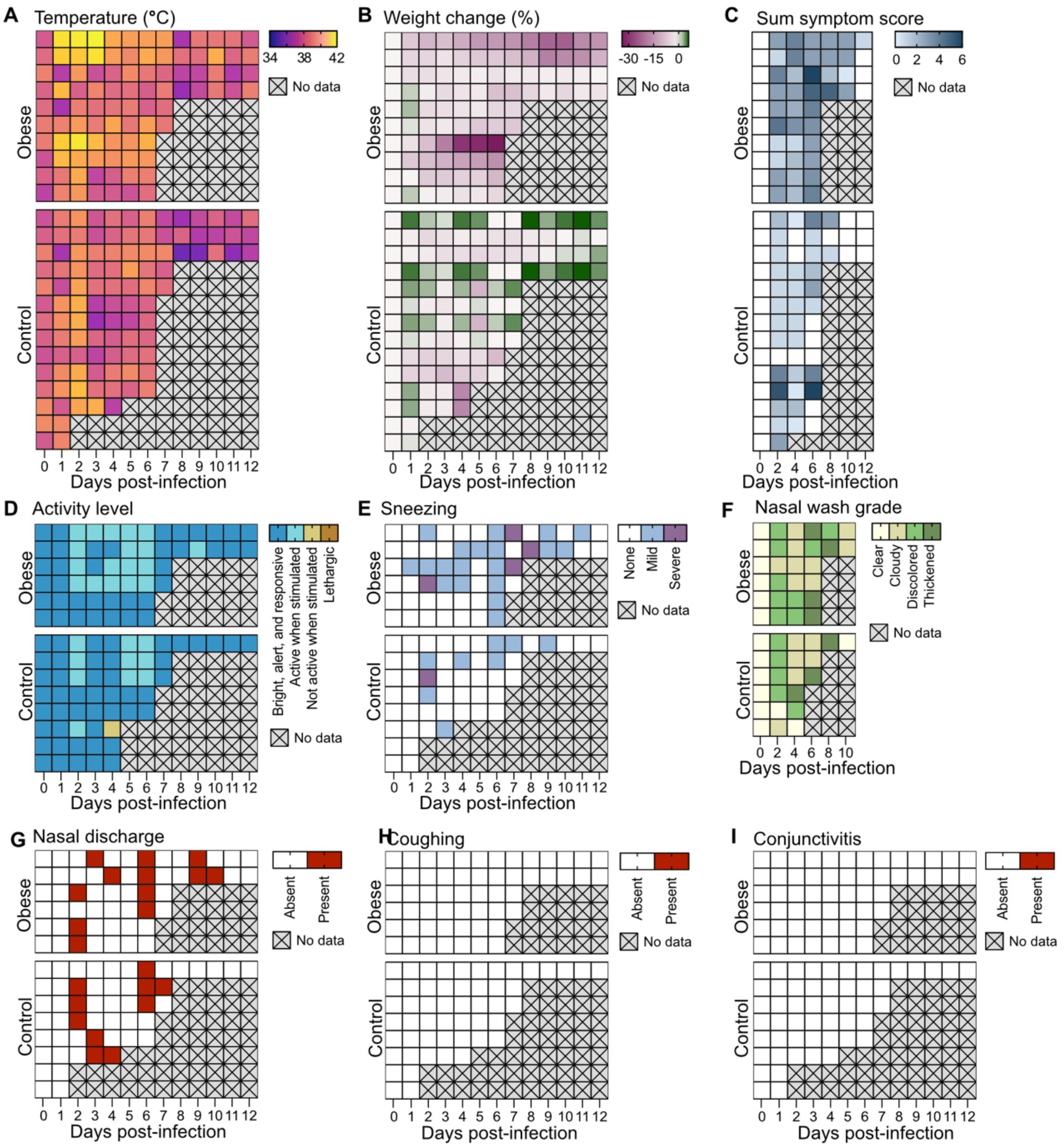

Fig. S3. Symptom log of H1N1 virus-inoculated ferrets. Obese (n=10) or control (n=14) ferrets were intranasally inoculated with 10^6 $TCID_{50}$ of A/California/04/2009 (H1N1) influenza virus and monitored for 12 days post-infection. Severity of (A) temperature, (B) weight change, and (C) sum symptoms are recorded for each ferret. Sum symptom score comprises (D) activity level, (E) severity of sneezing, (F) nasal wash grade, (G) presence of nasal discharge, (H) presence of coughing, and (I) presence of conjunctivitis. This data corresponds with Fig. 2C. Symptoms were recorded daily for (A-B, D-E, and G-I) and every other day for (C, F) due to nasal wash sampling schedule. Historically, ferrets display none-to-mild symptoms during infection with human influenza viruses so the literature reports weights and temperatures; however, due to the more severe symptoms observed in the obese ferrets we expanded our clinical scoring scale. Therefore (D-I) are reported for only n=6 obese and n=6 or 8 control ferrets per diet group.

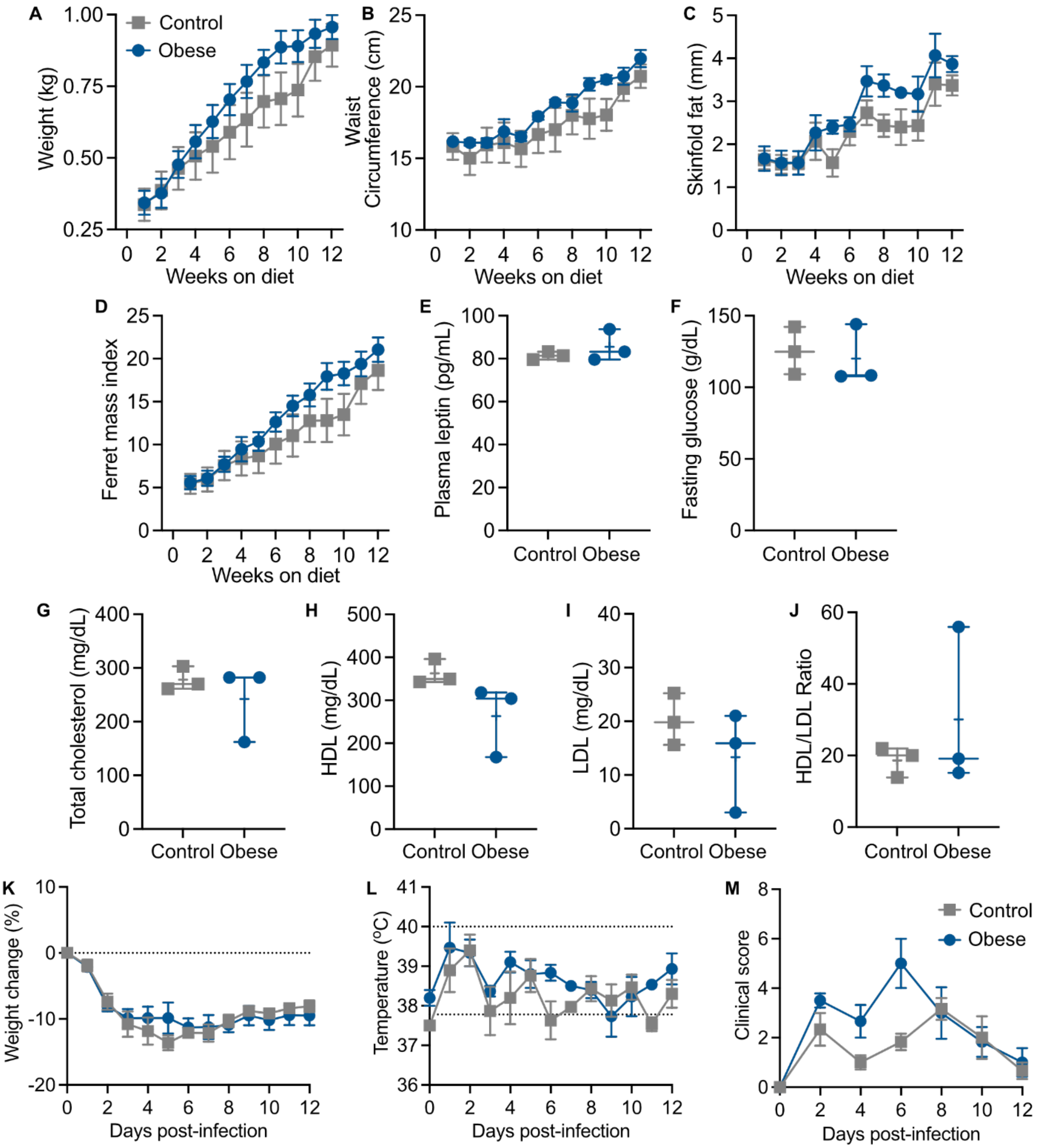

Fig. S4. Obesity and disease severity in female ferrets. Female ferrets (6 weeks of age) were placed on control or obese diet and monitored for 12 weeks. Weekly measurements were taken of (A) weight (p=0.4308), (B) circumference (p=0.3888), and (C) skinfold fat (p=0.2389). (D) Ferret mass index was calculated from physical measurements (p=0.3995). Data was analyzed by 2-way ANOVA with repeated measures (p-value represents simple main effect of diet). (E) Leptin (p=0.3987), (F) fasting glucose (p=0.7424), (G) total cholesterol (p=0.4396), (H) high-density lipoprotein (HDL) (p=0.1222), and (I) low-density lipoprotein (LDL) (p=0.3166) levels in the plasma of female ferrets post-diet. (J) Ratio of HDL to LDL (p=0.4401). Data shown in E-J was analyzed by unpaired t test. (K) After 12 weeks on diet, female ferrets were infected with 10^6 $TCID_{50}$ A/California/04/2009 (H1N1) virus and monitored for 12 days. Weight (p=0.9096), (L) body temperature (p=0.3400), and (M) clinical scores (p=0.1703), were recorded throughout infection. Significance was determined by 2-way ANOVA with repeated measures (p-value represents simple main effect of diet). Data represents 1 independent experiment of n=3/group.

Error bars are standard deviation of the mean (SD) and dashed lines indicate baseline weight (K), normal range (L) or lower limit of detection (M). *$p<0.05$, #$p<0.10$.

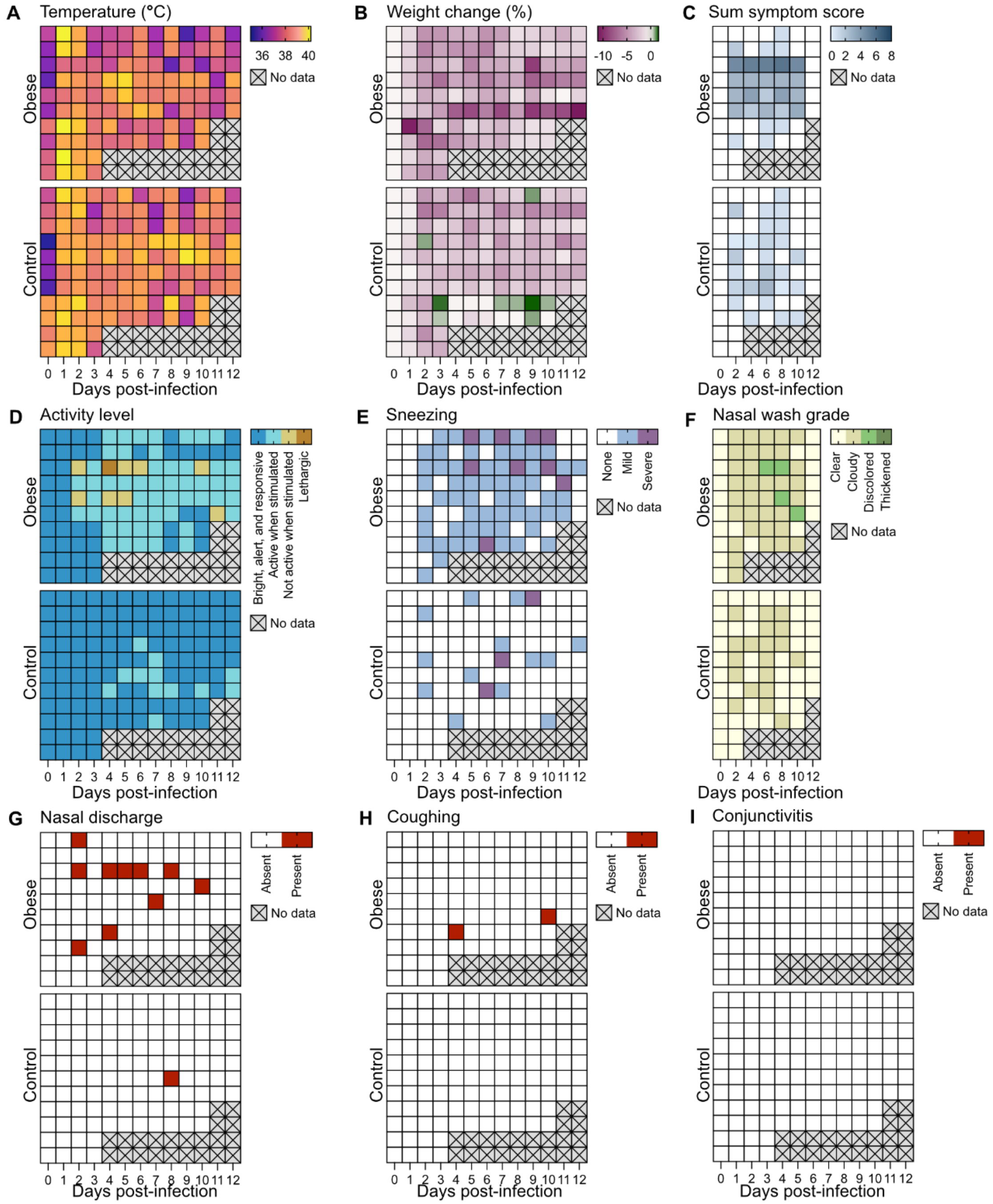

Fig. S5. Symptom log of H3N2 virus-inoculated ferrets. Obese (n=10) or control (n=11) ferrets were intranasally inoculated with 10^6 $TCID_{50}$ of A/Memphis/257/2019 (H3N2) influenza virus and monitored for 12 days post-infection. Severity of (A) temperature, (B) weight change, and (C) sum symptoms are recorded for each ferret. Sum symptom score comprises (D) activity level, (E) severity of sneezing, (F) nasal wash grade, (G) presence of nasal discharge, (H) presence of coughing, and (I) presence of conjunctivitis. This data corresponds with Fig. 4A, D, G. Symptoms were recorded daily for (A-B, D-E, and G-I) and every other day for (C, F) due to nasal wash sampling schedule.

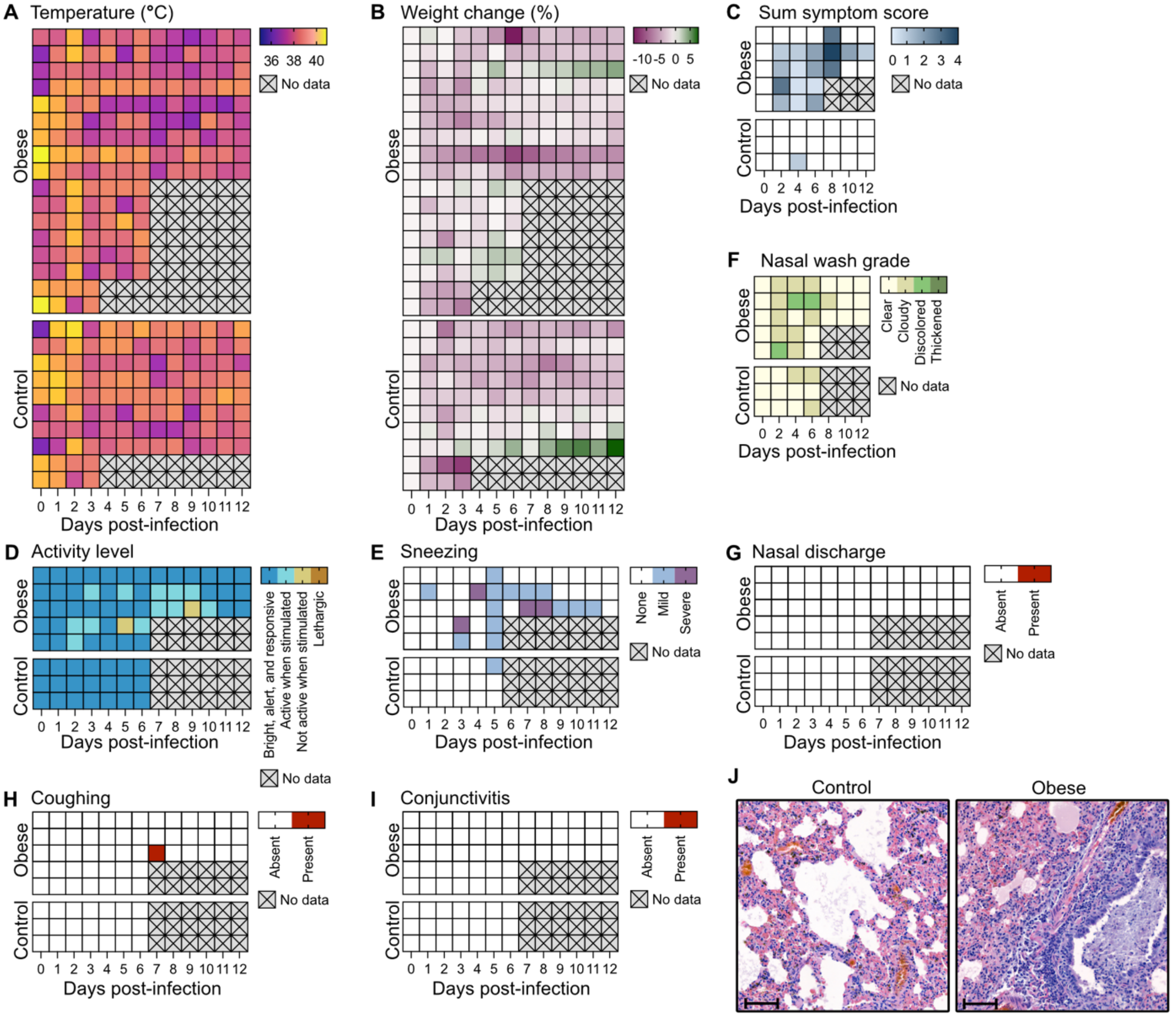

Fig. S6. Symptom log of H9N2 virus-inoculated ferrets. Obese (n=17) or control (n=10) ferrets were intranasally inoculated with 10^6 $TCID_{50}$ of A/Hong Kong/1079/1999 (H9N2) influenza virus and monitored for 12 days post-infection. Severity of (A) temperature, (B) weight change, and (C) sum symptoms are recorded for each ferret. Sum symptom score comprises (D) activity level, (E) severity of sneezing, (F) nasal wash grade, (G) presence of nasal discharge, (H) presence of coughing, and (I) presence of conjunctivitis. These data correspond with Fig. 4B, E, H. Symptoms were recorded daily for (A-B, D-E, and G-I) and every other day for (C, F) due to nasal wash sampling schedule. Historically, ferrets display none-to-mild symptoms during infection with human influenza viruses so the literature reports weights and temperatures; however, due to the more severe symptoms observed in the obese ferrets we expanded our clinical scoring scale. Therefore (C-I) are reported for only n=5 obese and n=3 control ferrets. (J) Hematoxylin and eosin staining of lungs taken from ferrets infected with 10^6 $TCID_{50}$ A/Hong Kong/1079/1999 (H9N2) virus at 6 dpi. Data represents 2 independent experiments of n=2/group with 5 histology

sections per ferret. Scale bar is equal to 100 μm. Data represents 2 independent experiments with n=2/group.

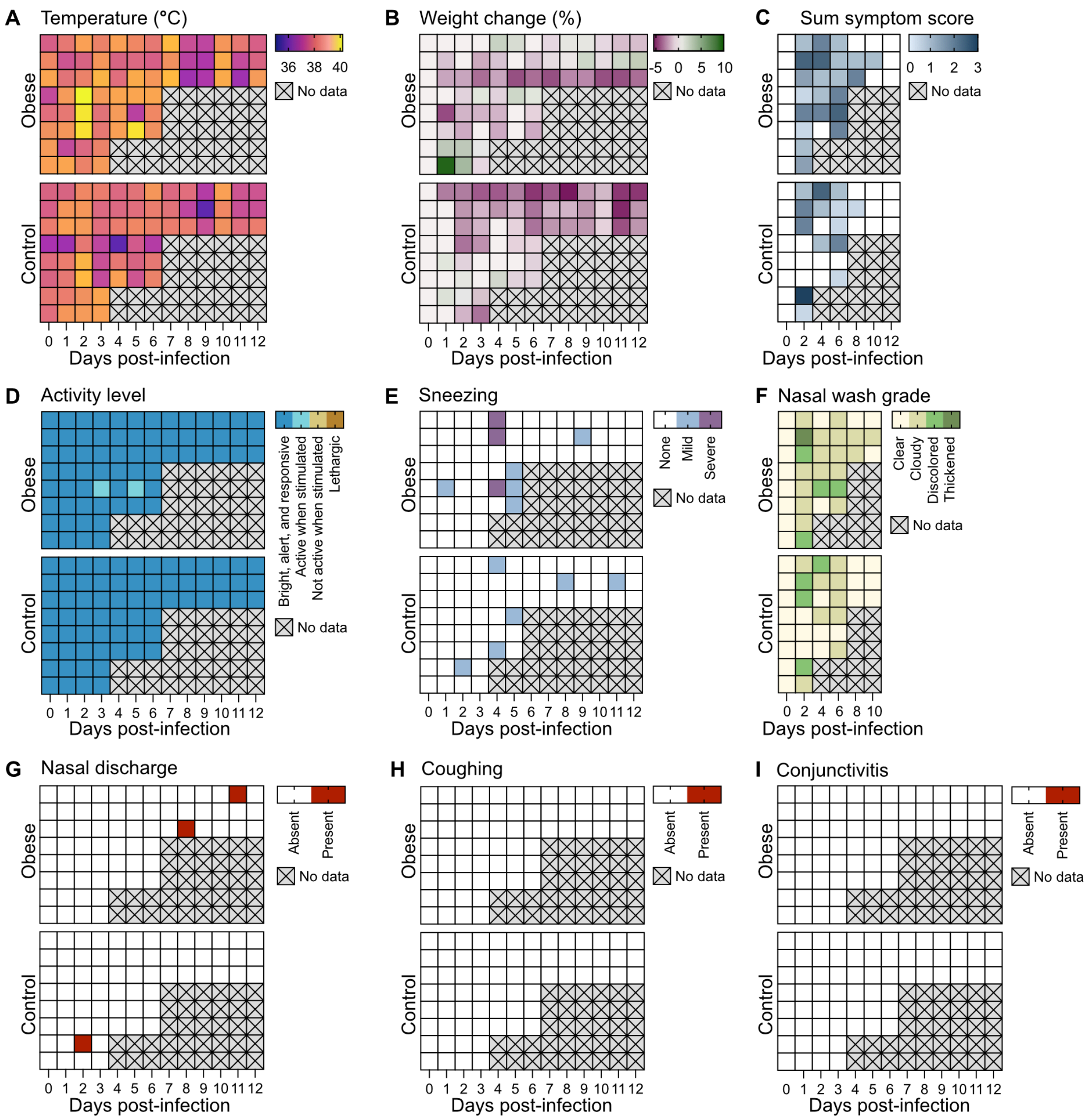

Fig. S7. Symptom log of influenza B virus-inoculated ferrets. Obese (n=8) or control (n=8) ferrets were intranasally inoculated with $10^{5.5}$ $TCID_{50}$ of B/Brisbane/60/2008 influenza virus and monitored for 12 days post-infection. Severity of (A) temperature, (B) weight change, and (C) sum symptoms are recorded for each ferret. Sum symptom score comprises (D) activity level, (E) severity of sneezing, (F) nasal wash grade, (G) presence of nasal discharge, (H) presence of coughing, and (I) presence of conjunctivitis. This data corresponds with Fig. 4C, F, I. Symptoms were recorded daily for (A-B, D-E, and G-I) and every other day for (C, F) due to nasal wash sampling schedule.

Table S1. Diet-induced obesity feeding plan per ferret

Weeks of age	Weeks on diet	Diet specifics		Metabolizable energy (kCal)	
		Obese	Control	Obese	Control
6	0	67.5 g ground ferret + feline diet<br>+7.5 g milk replacer<br>+ 87.5 mL distilled water*	45 g ground ferret diet<br>+ 5 g milk replacer<br>+ 50 mL distilled water	244.7 + calories from milk	187.2 + calories from milk
7	1	67.5 g ground ferret + feline diet<br>+ 87.5 mL distilled water	45 g ground ferret diet<br>+ 50 mL distilled water	244.7	187.2
8	2	45 g ground ferret + feline diet<br>+ 50 mL distilled water<br>67.5 g ferret + feline diet	30 g ground ferret diet<br>+ 30 mL distilled water<br>30g ferret diet	288.7	249.6†
9	3	*Ad libitum* ferret + feline diet	45 g ferret diet	288.7+	187.2
10+	4+	*Ad libitum* ferret + feline diet<br>37.5 g wet kitten food	45 g ferret diet	334.1+	187.2

*For gruel fed from weeks 0-2, additional water was needed due to higher carbohydrate content of the obese diet.

†Increases in estimated calorie content during week 2 on diet due to transition from gruel to solid food.

Table S2. Primer sequences

Gene	Forward primer	Reverse primer
Ace	CCAACCTGCCTGTGGAAAA	CCTGGCTGTGGAGTAGATCC
Adipoq	GAGATGGCACTCCTGGTGAA	GGTTTCACCAGTGTCACCCTTA
Areg	CGAGTTGCCCCAGAGACC	TGACATCCAATCCAGCAGCATA
Atf4.C	TTTACCTTCCTGCAACCACTTC	TCATGGTAATGTAAGCAGTAGAGTC
Ccl2	AAGAGAGTCACCAGCAGCAA	TCAGCGCAGATCTCCTTGTTA
Cd8a	CTGAGCAATTCGGTGCTGTAC	CGTAGGGGTGGGCTTGAC
Crp	CTGCAGACACACGCAGAAA	AGCCTTCAGTGGCTTCTGTA
Edn1	CTCTGCTGTTTGTGGCTTTCC	TCCCCTCCGCTGTCCAA
Egf	ATGGAGGTTCAGTCCATTTTAGC	GGGACATTGCAAACAAAGTGTG
Egfr	TGGTCCTTGGGAATCTGGAAA	GTGTTCAGGGCAATGAGGAC
Fas	GCAACTGCTGTCAACTCCAA	TGCAGTCACCCTCCTTCC
Gapdh.c	TGCGGCCAAGGCAGTAG	AGGCCATGCCAGTGAGCTT
Gstcd	CTGACCCCTCCAAGAAGGAA	GGATGGTAAGTTCGCATAGCC
Gzmb	CCACTCCTGCTCCTGTTCC	GCCATGTAAGGCCGGGAA
Hprt.c	CACTGGGAAAACAATGCAGA	ACAAAGTCAGGTTTATAGCCAACA
Icam1	CGCTCCTCGCCCTGAT	GCACTGATATTTGGGCATCCC
Ifitm5	CGCTGGCCTACTCCATCAA	TTGGCTTTGGACCCCAGAC
Ifna.c	TCCATCCTGAGGAACTACTTCCAG	AGGCACAAGGGCTGTATTGC
Ifnar2	TCGATTGTACCAGATCGCTATACA	GACCTCCCACACATCCGTTA
Ifnb1	GGTCACCCTTGAGATGTTCCA	AGGTTCTCGACAATGCTCTCA
Ifne	CTGAAACTGGCTCTCTTCCAAC	GACTGCTGGGAAAGGAGGAA

Ifng.c	AACTGGAGAGAGGAGAGTGACAAAA	GTCTTCCTTGATGGTATCCATGC
Il1a	TCACTGATGATGACCTGGAAGT	CGGAAGTTGTATGCTGCAGATC
Il1b.c	CCTGGTGCTGTATAACTCGTATGAG	TTGGTTCACACTAGTTCCGTTGA
Il1rl1	AAGTTCCTCCCTGCCAAAGT	ATACCCAGTCTTGTTGGAGGTC
Il2	TTCGCAAACAGTGCACCTAC	CAACTGTAAGTCCAGCAGCAA
Il2ra	TTCATGCGTTGTGCAGGAAA	CTCTGCATTGCCTTCTCTTCC
Il4	TCACCGGCACTTTCATCCA	ACGGTCAGCTCCATGCA
Il6.c	GCAGAGAACAACCTAAATCTTCCAA	TGATTGAATTGAGACTGGAAGCA
Il7r	CCCGACGTCAATACCACCAA	CGCTGTCTCCGATGAGTAAGAA
Il8	GCTTTCTGCAGTTCTGTGTGAA	AGGCGTGGAGTGGGTTTTAA
Il9	GTCCGACCTTCATAGGGATCA	GGCTGCAGTTGCATTTTGAA
Il10	CCTTCGGCAGGGTGAAGAC	CCTTCGGCAGGGTGAAGAC
Il12a	CTGTCAGCAACACGCTTCA	CCACGGTGCTGGTTTTATCC
Il17a	CGGGAATACGAACTCCAGAAGA	GGGGTATCTCTCAGGGTCCT
Il17f	CAAATTTCCAGAACCGCTCCA	CGCAATCTCCGAGGGGAA
Il33	CCAAAAGGCATTCCCCAAGAA	CTGCTGATGACAAGCAGTGAA
Irf1	GAGGTTGAGCGGGACCTAAC	CACTGGGATGTGCCAGTCA
Irf2	AGATGCGCCCCTCTTTAGAA	GCCTTCCATGTTTTGGGATCA
Irf3	GAAGACTTCGGCATCTTCCA	AGGTGGGCAGATCAGGTTTA
Itga1	CGGTGGAGGACATGTTTGGATA	CCCAACTGGACACTTGTAGACA
Itga2	TGCAGTGCAGCAGTTTGTAA	TTCTCAGGAAAGCCACTCCA
Itga3	GCGGCAGCAGCGTTAC	CCGGTTGGTGTAGCCATCA
Itgb2	CTGCGCCTGGTGTCAGAA	TCGCAGCGAACAGAGTCC

Itgb5	CAAAATGTGCCTGGTGCTTCA	TGGCCAGGTTTGCCTTCAA
Itgb6	CAGTGTGCCTGGTGTTCTCA	AGAGTTGCTGGGGTATCACAC
Mmp1	GGAGCACACCCACCTGAC	TGTCCACGTCTGCTCTTGAC
Mmp2	GGCCAACTACAACTTCTTCCC	CGGTGTGTAGCCAATGATCC
Mmp3	TGGAGGAAAACCCACCTTACC	CAGAGACTCAGGGCTTTCTCAA
Mmp7	CGCCTGGTGGAAATAATGCA	ACGAGTTGATTGACGGTGAC
Mmp8	TCATGGTGACCCCTGGAAAC	CCTCTGACAACTGTGTGGTGTA
Mmp9	CCGGGAGATTGGAGAGCTAA	CATCTCGCCCACGAGAGTATA
Mmp13	ACGATCTGTCCGAGGAAGAC	CAGCAGGATTCATGGGGTAGTA
Mmp14	CTGTGACGGTAACTTCGACAC	ATTCCTCACCCTCCAGAACC
Mmp15	AGTGACATCAGTGCTGCCTA	GCTTCTCGGAAGAGCCAGTA
Mmp25	AGCCCTCCGCTCAGGA	TGGTGGCAGATAGCCATAGC
Mpo	CTAAGCCTGCTGGAGGGGAA	TTCAGCTGGGCTGGTGTCA
Mx1	CTCGGAGGTGGAAGCAGAA	GACTGATCCCCTGTCCTTCC
Nos2	CCCAGATGAGCTTCTACCTCAA	TCACCCTCACGTCCTGGTA
Retn	TGGACAAAGCCATCAGTGAGAA	GGTCAGGCCAAAGTTCCTCA
Sell	TGGAGATGTCACGGCTTTCA	TGGAGATGTCACGGCTTTCA
Serpine1	CAAGAGGCTATGCGGTTCCA	GCCCCATGAGCTCCTTGTAC
Serpine2	CGGTGATGAGATACAGCGTGAA	AAGCCGTTCTTCACGAACAC
Sftpb	AGTCACACTTGCTGCTGTG	ACGAGGCAGTCCAGTCAG
Sftpd	CTGCACCCTGGTCATGTGTA	TCTCCCTACAGCTCCTGGTAAA
Smad2	ACCACTACCAGAGAGTTGAGAC	GGCAGCTCTGTTAGGATCTCA
Smad3	ATCTTCTCTTGCCCCGAAGAA	CCCCTTCCGATGGGACAC

Stat3	GCTGCTTGGATCGAGAGTCA	ACCAAAGTCGCATGTGATTCC
Stat4	TCTGTCTGGTTTCGGGAGAC	GTTGGACTTGATTCCACTGAGAC
Tbx21	ACCACCTGTTGTGGTCCAA	TCCCGCCACAGTAAATGACA
Tgfb1	GCGTGCTAATGGTGGAAACC	GGAGCTCCGATGTGTTGAA
Tgfb2	ACATGCCGCCCTTCTTCC	TTCTTCTCCATCGCCGAGAC
Tgfbr2	CTCCGATGAGTGCAATGATCAC	TCAGGGTTGTTGGTGGCATA
Timp1	CGCAGCGAGGAGTTTCTCA	GTTCCAAGGAGCCACGAAAC
Timp2	ACCCCATCAAACGGATTCAGTA	GAAGGGGCCGTGTAGATGAA
Timp3	ACAAGTACCAGTACCTGCTGAC	CTCCACAAAGTTGCACAGTCC
Tradd	TCTGCGGACTGCACTGAC	GAAACGCACCTGCACAATCA
Traf1	CACATCCTGAGCTTGGAACAAC	CAGAGGAAGGTGCCATCGAA
Vcan	ACCGCTGTGACGTCATGTA	TCGCCGCTCTGTAGTGAAA
Vegf	ATTGGAGCCTTGCCTTGCT	TGTGCTCTCCTTCTGCCATG
Vim	GAGACAGGTGCAGTCCCTCA	TCTGGCGTTCCAGAGACTCA
Vwf	GGGCTCTGTGGCGACTTTA	TCATAGGGGTCCGAGGTCAA
